# Cancer classification with radiomics in controlled preclinical models

**DOI:** 10.1038/s41598-026-37757-8

**Published:** 2026-01-29

**Authors:** Kyle Drover, David A. Simon Davis, Katharine Gosling, Jason Price, Naomi Otoo, Ines Atmosukarto, Kylie Jung, Hany Elsaleh, Farhan M. Syed, Benjamin J. C. Quah

**Affiliations:** 1 Irradiation Immunity Interaction Lab, Division of Genome Sciences and Cancer, John Curtin School of Medical Research, Australian National University, Canberra, Australia; 2 Division of Immunology and Infectious Disease, John Curtin School of Medical Research, Australian National University, Canberra, Australia; 3 Division of Genome Sciences and Cancer, John Curtin School of Medical Research, Australian National University, Canberra, Australia; 4 Radiation Oncology Department, Alfred Health, Melbourne, Australia; 5 Radiation Oncology Department, Canberra Hospital, Canberra Health Services, Canberra, Australia

**Keywords:** Radiomics, Cancer, Biomarkers, Medical imaging, Cancer, Image processing, Biomarkers, Preclinical research

## Abstract

The premise of radiomics involves extracting high-dimensional quantitative features from medical images to aid clinical decision-making. While radiomics has shown promise in predicting disease characteristics, concerns regarding confounders, reproducibility, and interpretability limit its clinical adoption. In this study, we assessed the ability of radiomic features extracted from contoured CT images to classify two distinct tumour models, CT26 colorectal cancer (CRC) and 4T1 breast cancer (BC), in a highly controlled murine setting. We aimed to provide compelling data for the role of radiomics as a reliable cancer biomarker. We benchmarked radiomics-based classification against previously established blood-based biomarkers, including leukocyte populations and plasma proteins. Feature filtering reduced the original 1409 radiomic features to 18 non-redundant, high-importance predictors, primarily texture-based transformations. Unsupervised clustering via UMAP revealed that radiomics-based features did not segregate tumour types as effectively as blood biomarkers, suggesting potential confounding factors. Supervised machine learning using Random Forest showed that radiomic features achieved a classification accuracy of 0.87, lower than the 0.96 and 0.99 accuracies obtained with cell and plasma biomarkers, respectively. Furthermore, integrating radiomics with blood biomarkers did not enhance classification performance, and feature importance analysis using SHAP identified blood-based markers as the dominant predictors. These findings suggest that while radiomics retains some predictive value, it is less effective than blood biomarkers in this classification task and does not significantly contribute to multimodal tumour classification models. Our study underscores the need for further standardization and validation of radiomics before its clinical implementation.

**Supplementary Information:**

The online version contains supplementary material available at 10.1038/s41598-026-37757-8.

## Introduction

Radiomics^1,2^ involves extraction of quantitative features, that are often indistinguishable visually, from regions of interest (ROI) in 3-dimensional medical images acquired by Computed Tomography (CT), Magnetic Resonance Imaging (MRI), or Positron Emission Tomography (PET) scans. The extraction is high throughput and has high dimensionality whereby hundreds of features can be extracted in minutes^3,4^. These features include shape features that describe the ROI’s overall geometry, intensity features that summarise the pixel densities in the ROI, and texture features that identify pixel intensity spatial patterns. Applying various mathematical transformations to image features can reveal additional information, often resulting in the generation of over a thousand distinct features^4^. The aim of generating these features is to gain insight into the underlying biology of the ROI to help with clinical decision making in conditions such as cancer^5^, cardiovascular disease^6^, lung disease^7^ and neuronal abnormalities^8^. As such, radiomics has been called a virtual biopsy^9^, referring to its potential to obtain similar information to tissue samples, but with the advantage of being less invasive. The process of using radiomics for predictions typically involves feature selection based on various statistical methods such as removal of non-variable and redundant features, coupled with supervised machine learning (ML) to make prediction. Several studies have reported on the capacity of radiomics to predict type and aggressiveness of cancers^10^ and even cellular^11,12^ and molecular^13^ characteristics of the underlying tissues. However, reports have also highlighted that uncontrolled confounders may lead to false or overvalued radiomic signatures in disease^14^. Radiomics methods also often lack standardisation, are difficult to interpret, and can be hard to reproduce, making it challenging to achieve widespread acceptance and apply them reliably at scale^15,16^. Furthermore, state-of-the-art diagnostic models, like the Clinical Histopathology Imaging Evaluation Foundation (CHIEF)^17^ model, are evolving with the rapid improvement in ML algorithms that achieve greater than 0.93 Area Under the Receiver Operating Characteristic Curve (AUROC) measures, meaning radiomics-based models must compete with this level of accuracy to be clinically useful.

We have an established record of using biological features such as blood cells and plasma proteins to make definitive supervised ML-based predictions on the presence and type of tumours in animal cancer models^18,19^. One of the key outcomes of these previous studies was the ability to predict if tumour-bearing animals had a CT26 model colorectal cancer (CRC) or a 4T1 model breast cancer (BC), with accuracy close to 100% across several studies. This gives us a unique benchmark with which to study the utility of radiomics in predicting the type of cancer present using ML models.

In this study, our aim was to assess how well radiomic features extracted from contoured CT images of CRC CT26 and BC 4T1 tumours were able to predict cancer type compared to the blood biomarker signatures we previously established. The rationale was to investigate whether radiomics could classify tumours in a highly controlled murine setting without confounders commonly seen in the human population. The mice were genetically identical, of similar age, housed and cared for in the same environment, had the same diet, and had tumour development in similar anatomical positions. Tumours of each class were genetically identical, scanning equipment and setting were identical, and tumours were contoured using the same standard operating procedure. The study also assessed if the combined use of radiomics and blood-based features improved predictive performance, and which specific features contributed most to predictions. In this highly controlled study, we aimed to generate compelling data for the predictive value of radiomics in defining cancer characteristics which, in turn, may guide future biomarker research across different modalities.

## Results

### Model establishment

**Fig. 1:**
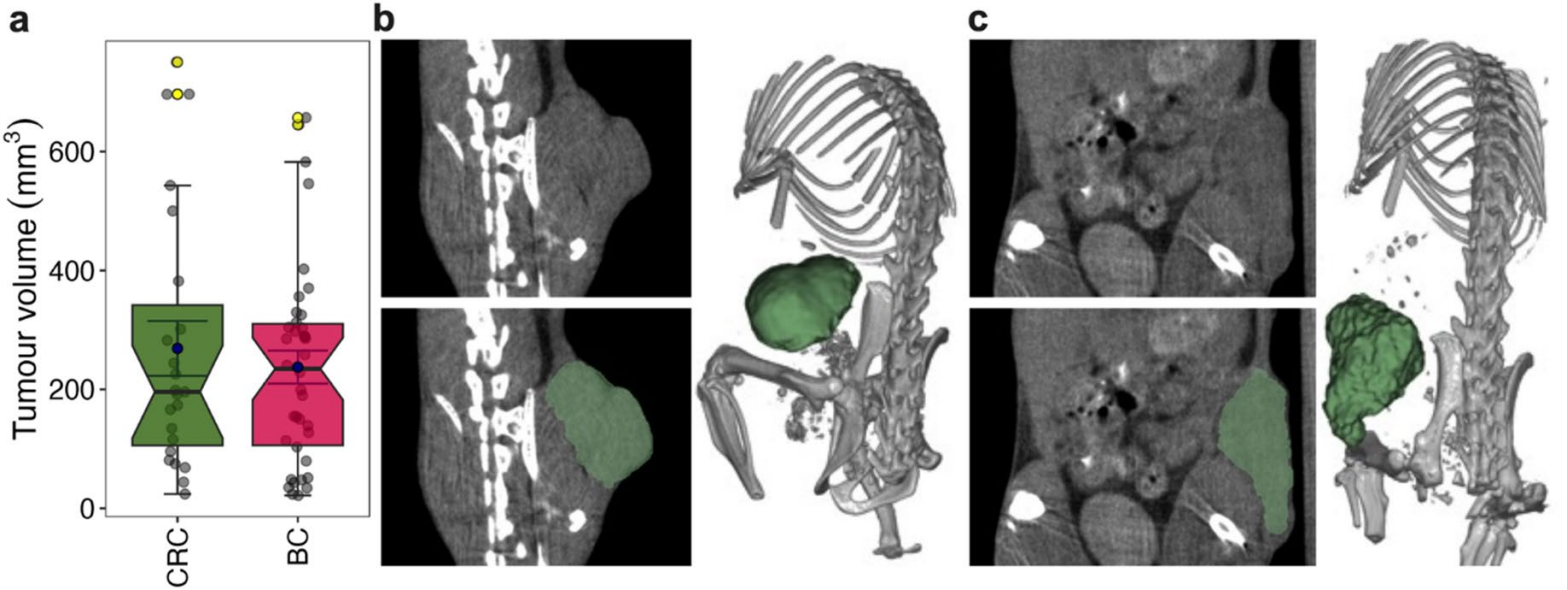
CRC and BC tumour sizes, scans and contours. CT26 CRC and 4T1 BC tumours were established subcutaneously in the right hind flank of BALB/c female mice and left to grow for 7–14 days. Tumour regions were imaged within these time frames using a micro-CT. Tumours were contoured in 3D Slicer using a semi-automated method and radiomic features extracted using Pyradiomics. Tumour contour volumes from radiomic features of the two classes across the imaging time points are shown in (**a**). Representative images of animals with CRC (**b**) and BC (**c**), show axial plane (left panel) and 3D reconstruction (right panel with bone thresholds) images as well as contoured tumour ROI in green. Plots in (**a**) are boxplots with notches representing 95% confidence intervals, blue points showing means, error bars (inner whiskers) being standard error of means, yellow points as outliers, and grey points individual sample values (CT26 samples = 34 and 4T1samples = 39). A Wilcoxon rank-sum test was used to compare tumour volumes between the CRC and BC groups. The test yielded a p-value of 0.713, indicating no statistically significant difference in tumour volumes between the two groups.

CRC CT26 and BC 4T1 tumours were established subcutaneously in the flanks of BALB/c mice. After 7 to 14 days, tumours exhibited a range of sizes (Fig. 1a). At these times mice underwent CT scans under anaesthesia, had their tumours contoured (Fig. 1b and c), and 1409 radiomic features extracted using the python package Pyradiomics^4^.

### Feature filtering

**Fig. 2:**
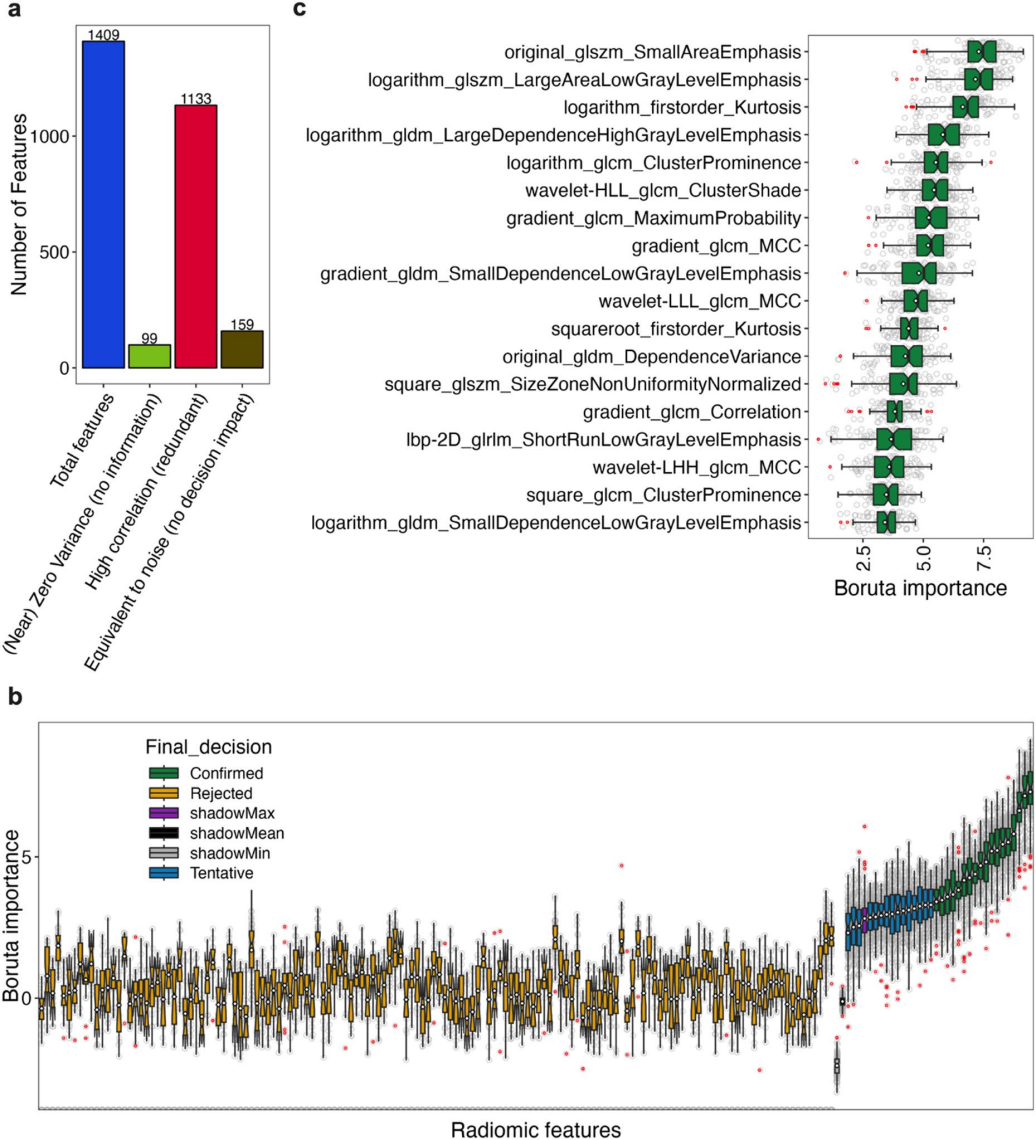
Radiomic feature filtering. Radiomic features from the entire CRC and BC dataset (CT26 samples = 34 and 4T1samples = 39) were assessed for zero variance (defined as all values in the feature being identical), and near zero variance (defined as having the frequency of the most prevalent value over the frequency of the second most prevalent value being > 19 and the unique values to total values being ≤ 10%). Features meeting these low variance criteria were removed ((Near) Zero variance (no information) in **a**). Features were also assessed pairwise for Pearson’s correlation coefficients of > 0.9 and, if meeting this threshold, the second paired feature was removed, and this repeated until all redundant features were removed (high correlation (redundant)) in **a**). Finally, remaining features were assessed to determine if they were statistically equivalent to shadow features (the equivalent to background noise and generated by shuffling the true features) for classification of CRC and BC using Random Forest in the Boruta algorithm, and if equivalent (rejected) or close to equivalent (tentative) were removed (equivalent to noise (no decision impact)) in **a** and **b**). Boruta also ranked the importance of the features based in the built-in Random Forest importance score (Boruta importance) (**b**). This left the features “confirmed” as being important which were used for the remainder of the study (**c**). Plots in **b** and **c** are boxplots with notches representing 95% confidence intervals, red points as outliers, and grey points as individual values.

Due to the large number of features generated, we first explored potential feature reduction by identifying features with low variance and features with high pairwise correlation that would indicate feature redundancy. We found that 99 of the 1409 features had zero or near zero variance across the samples, indicating that they contained little or no information and would not be useful in describing differences (Fig. 2a), and these were removed. We also compared the features for pairwise correlations that had a Pearson’s correlation coefficient of 0.9 or above, which would suggest a high degree of feature redundancy. A total of 1133 features were assessed as being redundant using this threshold and were removed (Fig. 2a). This left a total of 177 relatively non-redundant features that exhibited a variance above threshold. We sought to further filter these features based on their importance in a supervised classification task. To do this we used the Boruta algorithm^20^, which generates shadows of each feature by randomising their values to represent background noise. It then assesses if the feature was statistically better at making predictions using a Random Forest classifier^21^ relative to the shadow features, and ranks feature importance using the in-built Random Forest importance function. Using this approach, many features (159) did not contribute significantly to tumour classification and were equivalent to background noise (Fig. 2a and b). This left ~tens (18), rather than the original ~hundreds (1409), of features remaining after the filtering processes (Fig. 2c). The remaining selected features were mainly texture-based transformations, with few relating directly to intensity and none to tumour shape (Fig. 2c and Supplementary Table 1).

### Statistical analysis of selected features

**Fig. 3:**
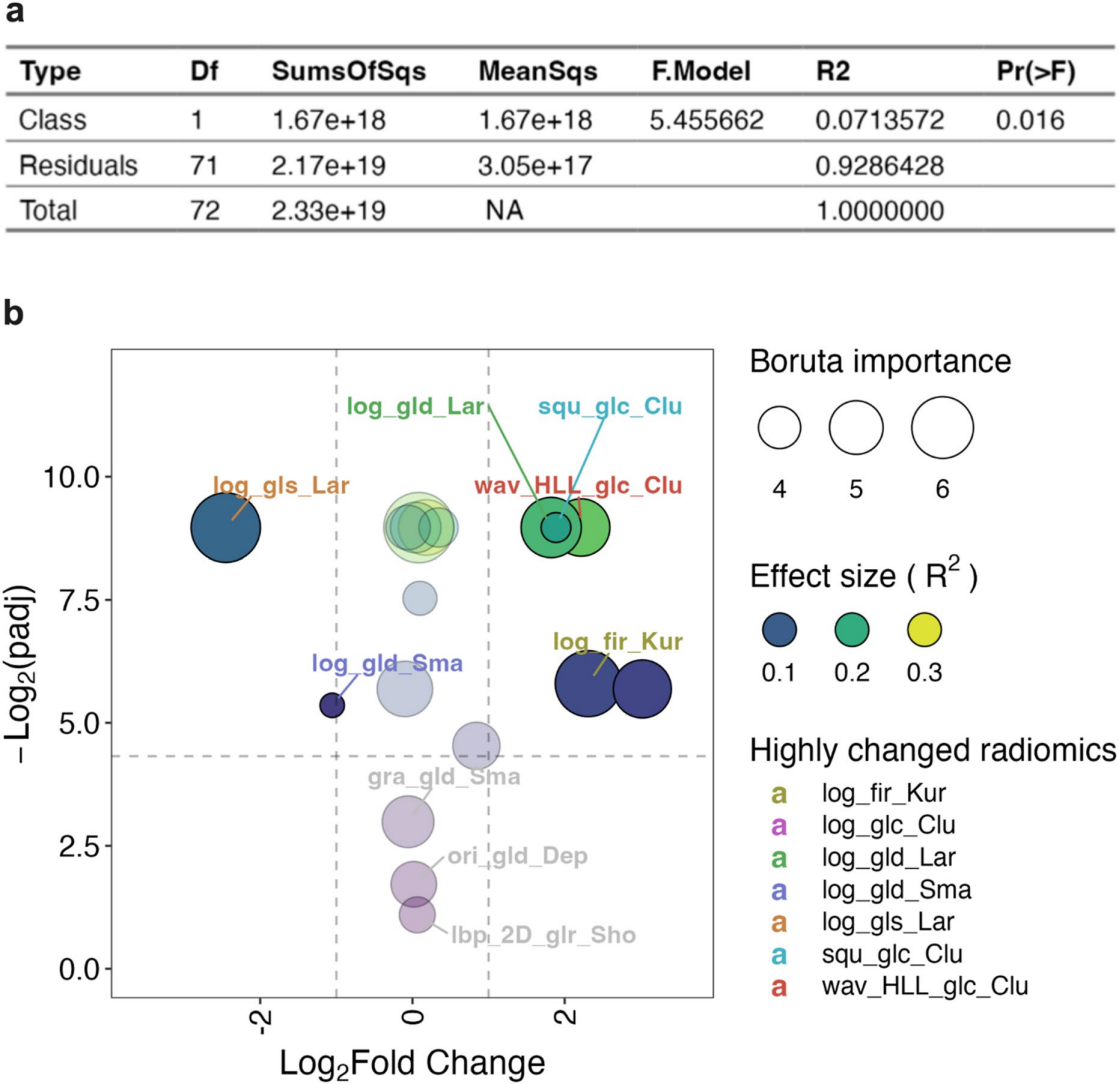
Multivariate and pairwise analysis of radiomics features between cancer types. Nonparametric Multivariate Analysis of Variance (PERMANOVA) was used to assess the effect size of cancer type in explaining data variance across all of the eighteen selected radiomic features (R2) and the statistical significance of cancer type in these differences (Pr(> F)) in a MANOVA table (**a**). Comparisons for each radiomic feature pairwise comparing BC to CRC was also performed using PERMANOVA and multiple comparisons for statistical significance corrected using FDR to give p adjusted (padj) values. The -log_2_(padj) was plotted against the mean fold change for each feature (Log_2_ transformed) and the pairwise effect size (R^2^) colours scaled for each data point; the most highly changed features are listed as truncated feature names (listed in full in Supplementary Table 3) (**b**). In addition, the Boruta importance was scaled as point size, a horizontal line place at -log_2_(padj) = 4.32 (equivalent to padj = 0.05), and vertical lines at Log_2_Fold Change = -1 and 1 (equivalent to a mean fold change of 0.5 and 2 respectively).

To determine if these features were collectively significantly different between CRC and BC tumours, we assessed them using a multivariate analysis of variance (MANOVA) approach. Since the data was not normally distributed or of equal variance (Supplementary Fig. 1), we used a non-parametric version of MANOVA (PERMANOVA)^22,23^ to assess whether the distribution of values significantly differed by cancer type across the selected features. This analysis revealed the feature values being significantly different was likely (having a p value of 0.016) and that the features were therefore different between the cancers (Fig. 3a). The analysis also revealed that cancer type explained 7.1% of the data variance (having a R^2^ = 0.071), suggesting moderate^24^ separation between the cancers and that other technical and/or biological factors play a more dominant role in data variance. To assess which features differed between tumour types, each feature was tested independently (Supplementary Table 2). The statistical significance of these differences (-log_2_(padj)) was plotted against the mean fold change in BC- versus CRC-derived radiomics features (Fig. 3b) in a volcano plot. Also indicated in the plot was the Boruta importance (point size), and the PERMANOVA size effect R^2^ (colour scaled) of each feature (Fig. 3b). Of the eighteen selected features, three did not appear to be significantly different (*p* > 0.05), and of the remaining fifteen significantly (*p* ≤ 0.05) different features, seven were highly changed (fold-change ≥ 2) between the tumour types (Fig. 3b). There did not appear to be a strong relationship between Boruta importance and size effect R^2^ or p values (Fig. 3b). Indeed, despite the three non-significant features being important for classification (via Boruta), comparing the raw data of these features between each tumour type clearly showed no differences in their overall distribution between the groups (Supplementary Fig. 2). When data variance was considered, the effect size (R^2^) revealed cancer type explained more than 15% (R^2^ > 0.15) of the variance of several features in a significant way, indicating a strong separation between the cancer types (Supplementary Tables 2 and Fig. 3b). The raw data comparisons also showed the highly variable nature of feature values and their distributions, and that they were a mixture of being higher or lower between the two classes (Supplementary Fig. 2). From this it appeared that several of the selected radiomics features were significantly and highly different between the tumour groups.

### Prediction performance against other cancer biomarkers

To benchmark the effectiveness of radiomic signatures in tumour class predictions, we compared them to cell and plasma biomarkers which we have previously found to be highly accurate in this classification task^18,19^. The cell biomarkers are based on quantification of several (16) blood leukocyte populations generated via antibody labelling and flow cytometry analysis, while the plasma biomarkers are based on quantification of several (23) proteins via LEGENDPlex analysis. The various biomarkers are listed in Supplementary Table 3.

#### Unsupervised ML

**Fig. 4:**
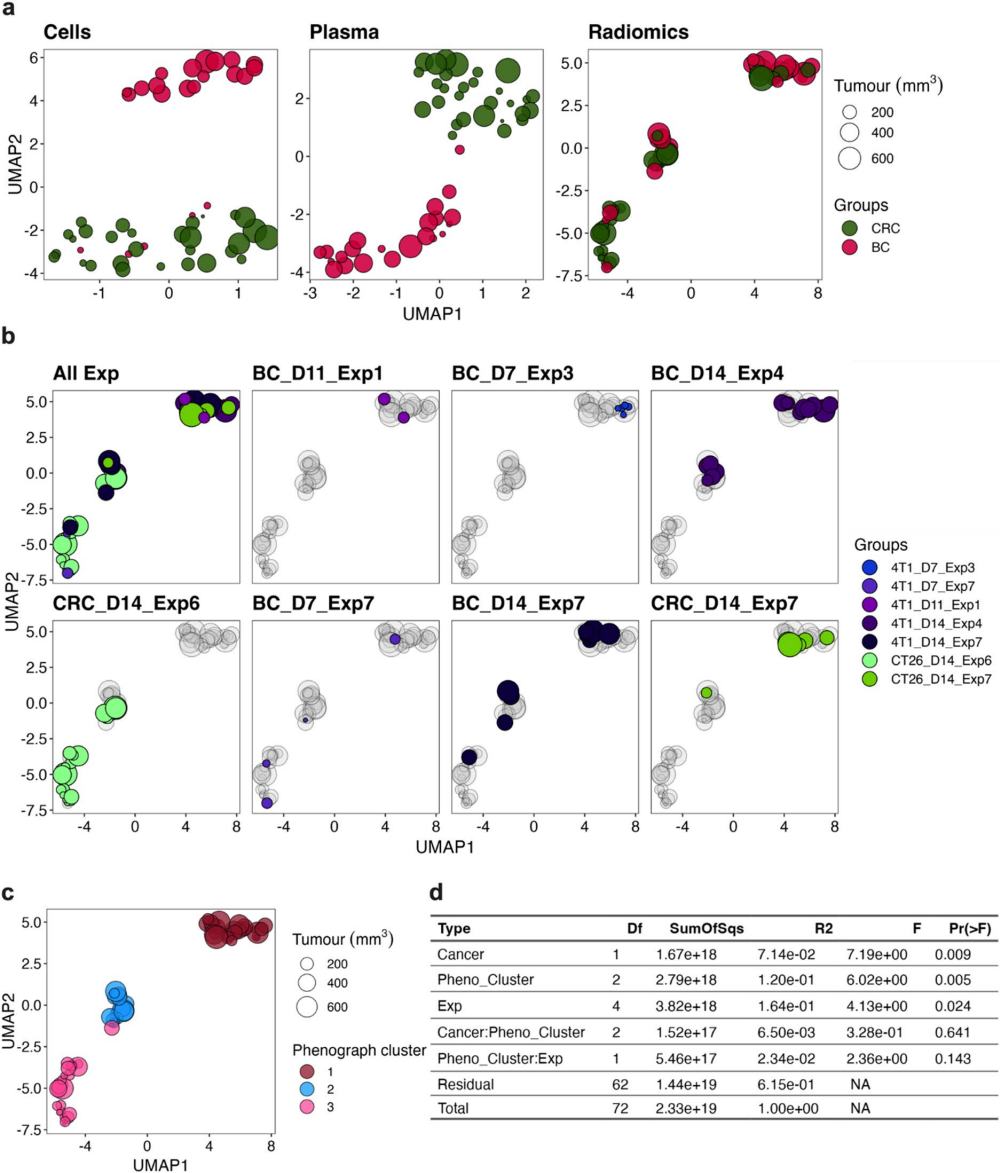
Unsupervised machine learning analysis using UMAP. Uniform Manifold Approximation and Projection (UMAP) was used to assess BC and CRC sample segregation using features derived from blood cells, plasma protein and radiomics from each animal (see Supplementary Table 3 for feature lists). Initial analysis looked at how tumour groups separated in the UMAP space across the three types of features (**a**) and subsequent analysis assessed how experimental times, annotated with tumour type (CT26 CRC and 4T1 BC), day (D7-14) and experiment number (Exp 1–7), segregated across the UMAP space (**b**). PhenoGraph was used to identify samples belonging to the same group based on the global data structure and the three clusters identified were mapped on the UMAP space (**c**). PERMANOVA analysis was performed to see how the PhenoGraph clusters (Pheno_Cluster) and experimental times (Exp) interact with cancer type in explaining the data variance (**d**). The blood cell and plasma data were downsampled to only include samples intersecting with the radiomic data set resulting in *n* = 29 for CT26 CRC and *n* = 22 for 4T1 BC. Radiomic data still had the original *n* = 34 CRC and *n* = 39 BC data samples. The point size in each plot scales with the corresponding tumour volume radiomic feature (i.e. original_shape_VoxelVolume).

Initially, we looked at unsupervised dimensional reduction using Uniform Manifold Approximation and Projection (UMAP)^25^ to assess the performances of all three feature sets in partitioning the two tumour classes in two dimensions (Fig. 4a). The cell-based biomarkers clearly separated two clusters in the UMAP space, partitioning the classes except for a small number of BC tumours that were of relatively small volume which overlapped with the CRC-predominant cluster. Although not as compact or discreet, the plasma-based biomarkers also produced two separate clusters for the cancer types and appeared to segregate the tumours more purely regardless of tumour volume compared to the cell-based biomarkers. In contrast, radiomic features appeared to form three clusters in the UMAP space which all contained a mixture of both cancer types. This suggests that the radiomic features may not be as effective as the cell or plasma biomarkers in tumour segregation, and/or potential uncontrolled variables may be confounding the partition. One such uncontrolled variable may be daily imaging fluctuations which can be visualised by mapping data points based on the day of experiment. Data segregation using this information showed no clear experiment-based clusters in the UMAP space, with tumour types partitioning over the three separate clusters even within the same experiment (Fig. 4b), thus suggesting that unknown variables may be impacting the cluster formation in a non-tumour specific way. To assess how the unknown variables may confound the radiomics’ performance on cancer type segregation, we clustered the data using PhenoGraph^26^, which uses k-nearest neighbour graphs, Louvain community detection and density-based refinements, to identify the three groups of samples clustered together by this dominant structure (Fig. 4c). We then reperformed PERMANOVA analysis using the PhenoGraph clusters (and experiment day numbers) as potential interacting variables with the cancer type to explain the variance seen in the radiomic features (Fig. 4d). This deeper analysis revealed that while the PhenoGraph and experimental variables explained a significant amount of data variability, their interaction with cancer type was not significant (Fig. 4d). Furthermore, cancer type still significantly contributed to data variance (although less so than experimental time and the PhenoGraph clusters) (Fig. 4d). This suggests that radiomics features may still have some utility in tumour classification if higher resolution ML approaches were used.

#### Supervised ML

**Fig. 5:**
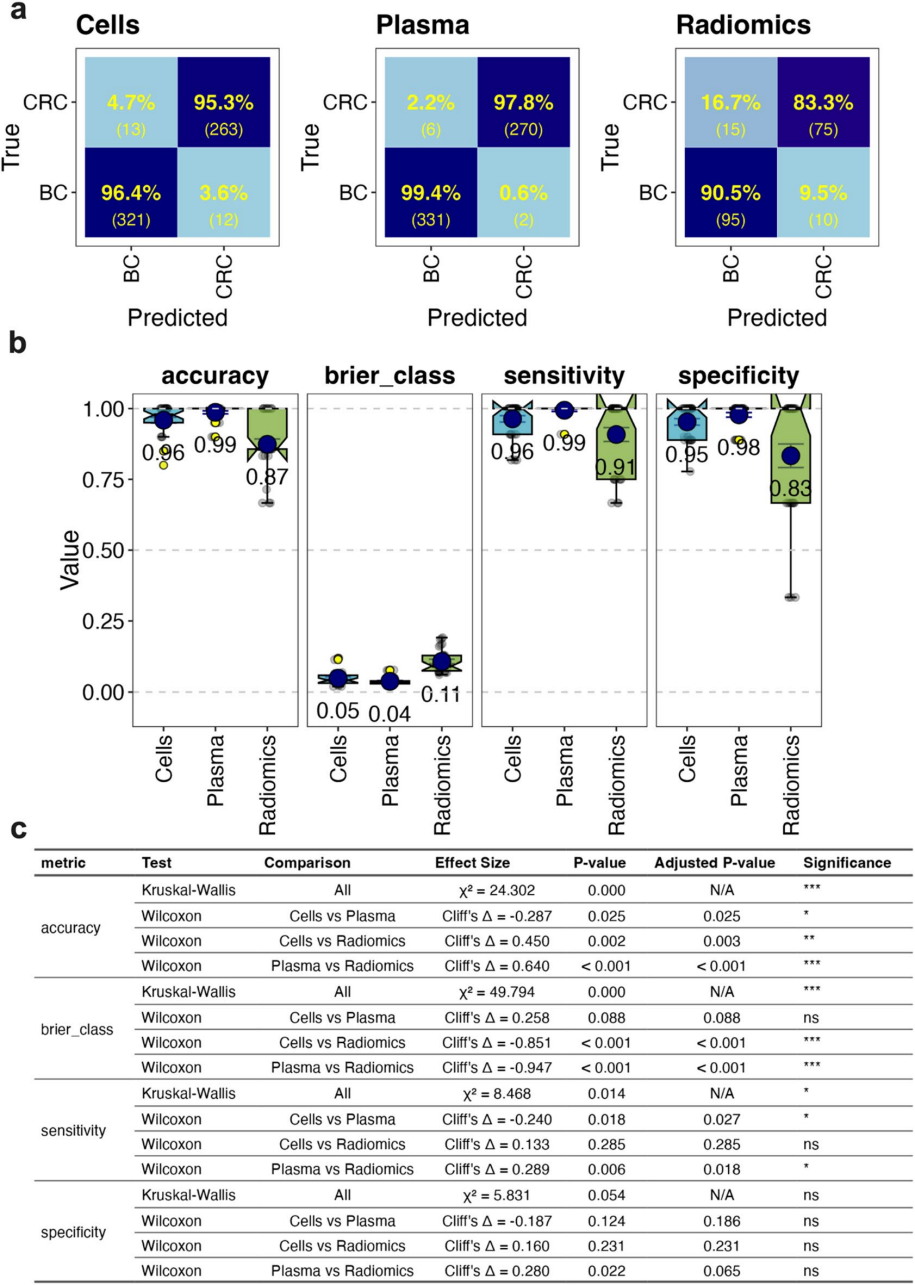
Supervised machine learning analysis using Random Forest. Random Forest was used to assess if BC and CRC classification differed when using features derived from blood cell, plasma protein and radiomics features (see Supplementary Table 3 for feature lists). A test set of four samples from each class was kept aside and the remaining training data used for initial analysis using 10-fold cross validation (CV) repeated three times. The training data sets, if required, were balanced to a dominant to subdominant class ratio of 1.2:1 or less by randomly removing dominant class samples. This resulted in the class numbers described in Supplementary Table 4 (Unimputed data). CV predictions of BC and CRC were depicted as confusion matrices that show the number predicted and true number of samples across all CV set repeats (in brackets) as well as the % of truth row-wise in colour scaled heatmaps (**a**). The accuracy, Brier class, sensitivity and specificity were also graphed as boxplots with notches representing 95% confidence intervals, blue points showing means (and shown also as text), error bars (inner whiskers) being standard error of means, yellow points as outliers, and grey points individual CV repeat values (**b**). Statistical analysis of overall differences (by Kruskal-Wallis test) and pairwise comparisons (by Wilcoxon test) for each feature type for each metric is also displayed (**c**). Adjusted P-values use FDR correction. The significance column is based on adjusted P-value (p.adj) thresholds: ns = not significant (p.adj ≥ 0.05), * = p.adj < 0.05, ** = p.adj < 0.01, and *** = p.adj < 0.001.

To further assess the utility of radiomic features in tumour classification, a supervised approach was used where learner algorithms were used to optimise predictive capacity on labelled data. We used a Random Forest learner^27^ and cross validation on a training dataset for initial assessment of classification performance^28^ (Fig. 5). Confusion matrices showed that cell and plasma-based biomarkers performed well in distinguishing BC from CRC with high true positive and negative rates (Fig. 5a and b), resulting in an overall accuracy score of 0.96 and 0.99, respectively, and low model uncertainty (as measured by Brier class) (Fig. 5b). The accuracy and sensitivity metrics were statistically different between the two blood biomarkers, (Fig. 5c) suggesting that plasma features are better at tumour classification than cell biomarkers. While radiomic features predicted BC with a high true positive rate, indicating high sensitivity for this class, this came at the cost of a higher rate of false positives for BC (Fig. 5a). Radiomic features also resulted in significantly less accuracy and model certainty than cell and plasma features (Fig. 5b, c) with an overall accuracy of 0.87 (Fig. 5b). Making the final models for each feature set on the whole training set and testing on the assigned test set produced similar trends across the cross-validation results (Supplementary Fig. 3), suggesting the models were not overfitting. Therefore, radiomics appeared to be inferior to blood-based biomarkers for cancer type prediction in this study.

Since the cell, plasma and radiomic feature sets were not fully overlapping with identical samples, we could not directly compare the relative importance of each feature set in the predictive performance in a combined model. Therefore, we imputed missing samples across the combined datasets using classification and regression trees in Multivariate Imputation by Chained Equations^29^. Combining the feature sets together in a partially imputed data set showed that radiomics did not significantly increase the predictive accuracy of the cell or plasma biomarkers either alone or together, and in many comparisons decreased prediction effectiveness (Supplementary Fig. 4a, b, and c), thus suggesting that radiomic features did not add to the utility of the blood biomarkers. The feature set combining all three biomarker types also allowed ranking of the importance of each feature across all biomarker types in collective predictions. To do this, we used Shapley Additive exPlanations (SHAP)^30^, which measures the contribution of each feature to the predictions made by the Random Forest classifier (Supplementary Fig. 5). The mean absolute SHAP values identified G-CSF (a plasma protein) and neutrophils (a blood cell) as the top contributors based on their relatively high SHAP values. The other top twenty features had more similar importance values and included a mix of cell, plasma, and radiomic features (Supplementary Fig. 5).

### Importance of contouring in radiomics

**Fig. 6:**
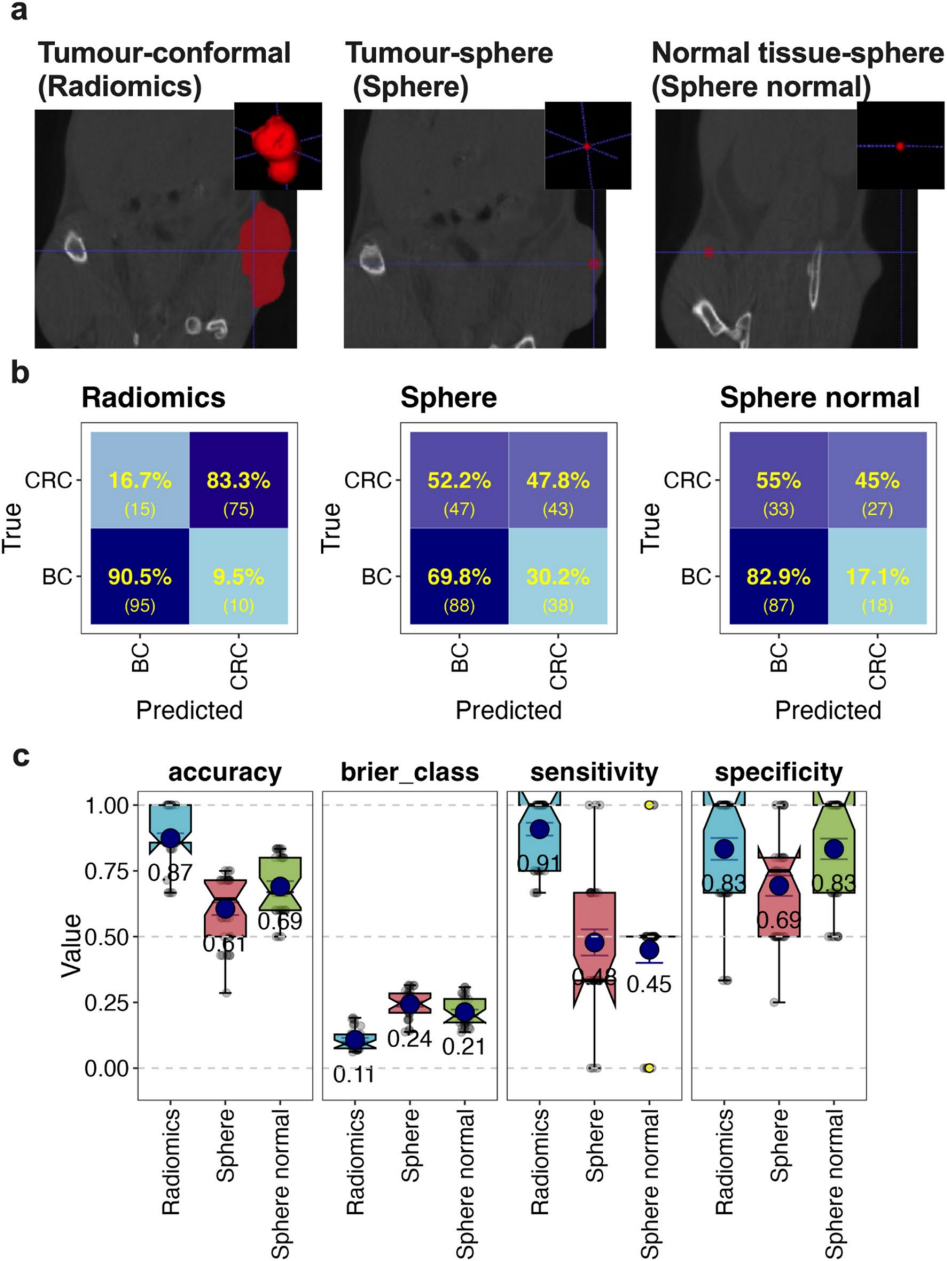

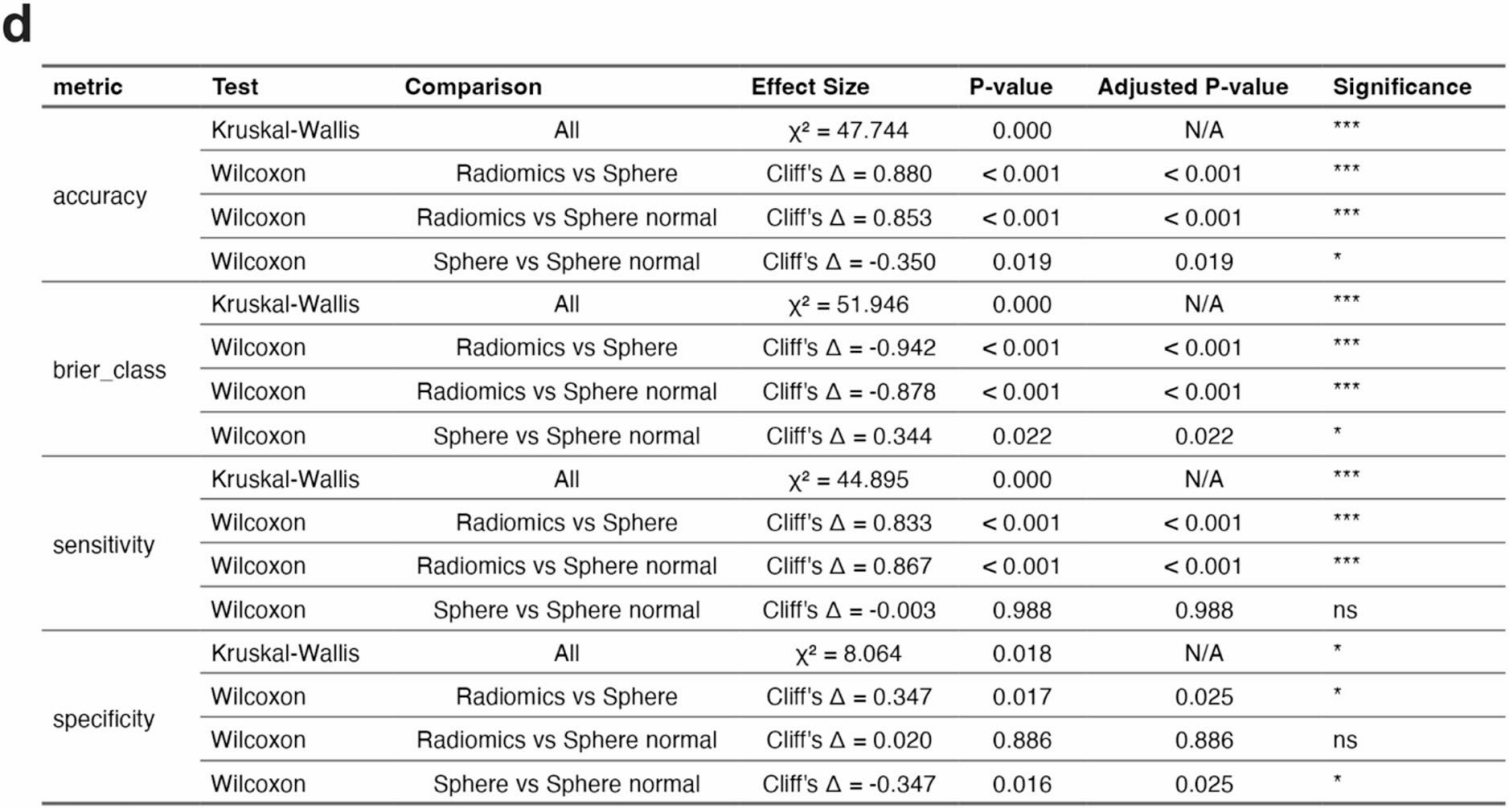
Using spheres to standardise ROI for radiomic feature generation. Random Forest was used to assess if BC and CRC classification differed when using the 18 radiomics features derived from different ROIs. The ROIs included conformal tumour contours (Radiomics), a 20-voxel diameter sphere (~ 1.2 mm) placed within the tumour volume (Sphere) or a 20-voxel diameter sphere placed within normal tissue contralateral to the tumour (Sphere normal) (**a**). Representative images of each type of ROI in (**a**) show the axial plane (main panel) and 3D volume (panel insert) with the ROI in red. The number of each class of tumour was balanced as described in Fig. 5 and sample numbers described in Supplementary Table 4. Analysis used repeated CV with predictions of BC and CRC depicted as confusion matrices (**b**) and performance metrics (**c**) as described in Fig. 5. Statistical analysis of overall differences (by Kruskal-Wallis test) and pairwise comparisons (by Wilcoxon test) for each feature type for each metric is also displayed (**d**). Adjusted P-values use FDR correction. The significance column is based on adjusted P-value (p.adj) thresholds: ns = not significant (p.adj ≥ 0.05), * = p.adj < 0.05, ** = p.adj < 0.01, and *** = p.adj < 0.001.

One of the challenges in using radiomics is the laboriousness of manual tumour contouring. In addition, considerable inter-observer variation (IOV) of contours can negatively impact on radiomic feature values^31^. We therefore aimed to assess whether a full contour was in fact required for the classification accuracy observed in our study, especially since the filtered radiomic features were not directly shape-based. We tested this by sampling the ROI using a sphere placed in the tumour instead of performing a full contour (Fig. 6a). Additionally, a control sphere of the same size was placed in the normal tissue of the same mouse on the contralateral side of the tumour (Fig. 6a). Supervised ML with cross-validation using the eighteen selected radiomic features from the spheres showed substantial confusion in classifying the tumours (Fig. 6b) with an overall accuracy of 0.61 for the tumour spheres and 0.69 for the normal tissue sphere (Fig. 6c). Most of the prediction accuracies, sensitivities and specificities were significantly lower than those from the full tumour contours (Fig. 6d). The spheres also resulted in significantly higher model uncertainty (Brier class) (Fig. 6d). This suggests that features from the full contour are required for optimal predictions and that these features are not homogenously distributed throughout the tumour.

## Discussion

Radiomics may improve clinical decision-making by offering detailed insights into the tumour region of interest (ROI). Its non-invasive nature may enable virtual sampling of tissue regions that are risky to access and longitudinal monitoring of lesions that might not be feasible with standard biopsies. Its utility will likely grow as imaging technologies become more sophisticated. However, there are many challenges in its implementation as standard clinical practice due to its multifaceted nature and the potential confounders and variability encountered therein^32^. These include inherent variability in patient biology such as age, sex, body mass composition, comorbidities and physiology which could impact on the appearance of imaging^33–36^. Radiomic features may also be impacted by technical variables such as specific scanner infrastructure, imaging reconstruction heterogeneity, contouring and post-processing variability, and motion artefacts^37^. Irrespective of whether these are true confounders (correlating with radiomic features and disease targets but not involved in causation) or introduce bias and/or variability, they may still have an impact on the prediction performance. Indeed, studies have highlighted the confounding effect of both biological and technical variables on radiomic outcomes^14^. Furthermore, considerable variability in predictive performance exists across radiomics studies that have used the same tumour type and research question^38–40^, highlighting the uncertainty of its reliability as a predictive marker. Our analysis, conducted under tightly controlled experimental conditions designed to minimize common confounders, provides several key insights into the strengths and limitations of radiomics for basic-level tumour classification.

Radiomics can yield many features, but the majority of these appear not to be useful in disease prediction. Our initial extraction yielded 1409 radiomic features; however, 99 of these contained almost no information, with zero or near zero variance, and the majority (1133) were highly redundant, with high pairwise correlations (Pearson’s *r* ≥ 0.9). Application of the Boruta algorithm distilled this set further to 18 non-redundant features—predominantly texture-based—with minimal contributions from intensity or shape parameters. This observation is in line with previous findings that there is a high degree of radiomic feature redundancy^41^ and that texture-based features are particularly informative for predicting tumour characteristics^42^. Indeed, it is well established that the breast cancer line 4T1 and the colorectal cancer line CT26 used in our study differ significantly in biological features that may drive such textural differences. CT26 tumours typically have less extracellular matrix deposition and stiffness but higher functional vasculature and blood volume compared with 4T1 tumours^43^. While both tumour types display tumour microenvironments dominated by myeloid cells and relatively low CD8⁺ T-cell infiltration, this imbalance is more exaggerated in 4T1^44,45^, consistent with CT26 being more responsive to immunotherapy than 4T1^46,47^. Many of the grey-level texture features in our filtered feature list overlap with the standardized set of 169 radiomic features proposed by the Image Biomarker Standardisation Initiative (IBSI) which were shown to be highly reproducible across multiple research teams using soft tissue sarcoma images^15^. Our study suggests that these IBSI features, and texture features more broadly, may be applicable to a wider range of tumour types. Refining radiomic feature sets to retain only the most informative, non-redundant texture measures may therefore enhance interpretability and efficiency, ultimately improving the translational utility of radiomics.

Despite our rigorous feature filtering process, our radiomics-based models only achieved an overall classification accuracy of 0.87 which was significantly lower than the 0.96 and 0.99 accuracies observed with our cell and plasma biomarkers, respectively. Furthermore, despite controlling for multiple sources of potential confounders, our unsupervised analysis by UMAP and Phenograph clustering revealed that radiomic features, unlike blood-based biomarkers, did not segregate tumour types cleanly; rather, they formed multiple clusters independent of tumour type. Since we standardised the methodology across the blood, plasma and radiomic biomarker pipelines up to the point of imaging and used the same scanner and contouring standard operating procedure for every sample for radiomics generation, we speculate that the dominant non-tumour clusters are driven by individual imaging fluctuations and/or inter-observer variability in tumour contouring, both of which are sources of variation well-documented in the literature^48,49^. Indeed, PERMANOVA analysis revealed that both experimental timing and time-independent variables identified through PhenoGraph clustering contributed significantly to data variance, potentially diluting tumour-specific signals in the images. These results imply that even in a controlled preclinical setting, technical variability inherent to image acquisition and/or processing may obscure tumour-specific signals in radiomic data. Despite this, these variables did not interact significantly with tumour type in explaining data variance, suggesting that tumour characteristics may be a significant contributor to the image difference between the tumour classes. To improve the utility of radiomics in tumour classification, we speculate that intra-image and/or intra-contouring normalisation may be crucial, possibly through the use of stage embedded standards and/or AI-assisted contouring to reduce variability. Even then, the problem of inter-subject variability, including differences in body composition, anatomical location of the tumour and imaging position, may be a barrier to the adoption of radiomics in clinical practice. While we used a subcutaneous site to control for tissue-of-origin effects, radiomic signatures may differ in orthotopic settings where tumour–host interactions play a larger role^50^, making site-specific analyses an important future direction to disentangle tissue- from tumour-specific features. A further limitation of our study is that our cohorts consisted of genetically identical, age- and environmentally- matched female mice bearing genetically identical tumours within each class type. While this design strengthens detection of tumour-specific signals, it does not capture the biological diversity of clinical populations. Future studies in more heterogeneous and clinically relevant models will therefore be needed to establish the robustness and translatability of our findings.

In our study, integrating radiomics with blood-based biomarkers did not yield improvement in overall classification performance of supervised ML models compared to the blood biomarkers alone. Furthermore, SHAP analysis revealed that the most influential predictors were blood-based markers—specifically, G-CSF and neutrophil counts—and while radiomic features did contribute their role was less important. This suggests the biological information captured by blood biomarkers may be sufficient for accurate tumour classification, and addition of radiomic features may have limited complementary value. This is perhaps unsurprising given the well-established role of immune cells and soluble immune mediators in tumourigenesis^51–53^. Further mechanistic insight into the relationship between tumour radiomics, the tumour immune landscape, and systemic immune changes could be achieved through single-cell RNA sequencing and flow cytometry–based profiling, enabling identification of immune and stromal populations underlying radiomic features. Complementary experimental approaches, including histopathological validation of tumour-infiltrating subsets and antibody-mediated depletion of key cell types (e.g., Ly6G-based neutrophil depletion^54^, could provide orthogonal corroboration of computational findings, thereby enhancing the biological interpretability and translational potential of radiomic biomarkers.

From a clinical perspective, radiomics provides a non-invasive “virtual biopsy”, spatially detailing tumour heterogeneity. However, our study demonstrates that even under tightly controlled preclinical conditions, radiomic features show lower classification performance compared to blood biomarkers, supporting existing evidence that technical variability, contouring differences, and other potential confounders must be addressed to improve reproducibility and clinical utility^14,33–36^. By directly benchmarking radiomics against blood-based biomarkers, we show that blood biomarkers not only outperform radiomics in classification accuracy but also dominate integrated models as key predictors. These results indicate that, while radiomics may offer complementary insights for longitudinal monitoring or inaccessible lesions, robust standardization of imaging acquisition, processing, and feature extraction—including intra-image normalization and AI-assisted contouring—will be essential to enhance reliability and clinical applicability. Overall, our findings provide experimentally grounded evidence that blood biomarkers currently offer more reliable and actionable information for clinical decision-making.

## Methods

### Animals

BALB/c female mice between 6 and 10 weeks of age were sourced from the Australian Phenomics Facility, Australian National University (ANU) and housed in a specific pathogen-free environment. Mice were cared for and used in experiments in accordance with conditions approved by the ANU Animal Ethics Committee under protocol A2020/39 and complied with the ARRIVE guidelines. All experiments were performed in accordance with relevant guidelines and regulations.

### Cell lines

The colorectal carcinoma CT26^55^ and the mammary carcinoma 4T1^56^ cell lines were sourced from American Type Culture Collection (ATCC) and cultured as previously described^19^ in supplemented RPMI-1640 (ThermoFisher Scientific).

### Tumour establishment

Tumours were established by injecting 1 × 10^5^ CT26 or 4T1 cells subcutaneously in the right hind flank of syngeneic mice mixed across housing cages. Tumour growth was monitored using digital calliper measurements over 7–14 days, with assessments performed in a blinded manner (score sheets contained de-identified row numbers). Growth trajectories and animal breakdowns, including the number of mice that underwent two CT scans (*n* = 5), are detailed in Supplementary Fig. 6. In total, 29 mice with 4T1 tumours were included, with 5 of these imaged at both day 7 and day 14 post-tumour establishment, yielding 39 4T1 tumour imaging samples. In addition, 34 mice bearing CT26 tumours were included, each imaged once, yielding 34 CT26 tumour imaging samples. Mice were ethically sacrificed by cervical dislocation at the study endpoint, which was determined by wellbeing and tumour burden scores, in accordance with the Australian National University Animal Ethics Committee protocol (A2020/39), or at a maximum of 21 days post-tumour establishment.

### Micro computed tomography (CT) scanning

Mice were imaged at days 7, 11 and/or 14 post-tumour establishment using a Quantum FX microCT (PerkinElmer). This involved anaesthetising mice with isoflurane using the in-built regulator and vaporiser, with sedation being induced and maintained with 3-3.5% isoflurane in oxygen. Once mice were fully sedated, tumour regions were imaged using a 4 cm square field of view under fine scan mode using 160 µA and 90 kV, resulting in 512 slices at ~ 80 μm thickness per slice. MicroCT image reconstruction and ring reduction were performed via inbuilt Quantum FX software (v2.2, PerkinElmer) and reconstructions were saved as Digital Imaging and Communications in Medicine (DICOM) files.

### Tumour contouring

DICOM files containing the mouse tumour microCT images were analysed using 3D Slicer v5.6.2^57^ (https://www.slicer.org). For conformal tumour contouring, window level and width were set to 1250 and 1110 respectively and segmentation performed with the segment editor module using a 1–3 mm paint tool. Tumours were initially contoured manually in the axial plane approximately 20 slices apart until 5 slices spread throughout the tumour were contoured and then the process repeated in the sagittal and coronal planes to give the overall structure of the tumour. The final contouring to create each ROI was then achieved using the grow-from-seed function followed by smoothing using the opening smooth method to exclude extrusions of 0.5 mm. In addition to conformal tumour contouring, sphere ROIs (~ 1.2 mm diameter) were also automatically generated using the Python raster_geometry^58^ package with smoothing enabled. Spheres were positioned at the midpoint of the tumour volume (referred to as Sphere), or in normal tissue on the opposite side of the animal; ~1.5 mm inwards to the mirror-inverse (z-axis) of the tumour (referred to as Sphere normal). The size of the sphere was set to keep within the minimum sized tumour (~ 1.2 mm width).

### Radiomics

A radiomics pipeline was created with Python (and documented in GitHub^59^ and used computing resources from the National Computing Infrastructure (NCI), ANU. DICOM files were converted to the required NRRD format using the C + + dcm2niix library^60^. Image intensity subtraction normalisation was performed by subtracting the differences in mean intensities between the sample stage and a specified reference stage across the entire image^61^. Radiomic features were calculated for each ROI using Pyradiomics v3.0.1^4^ with default settings used except that all features and filters were enabled. As such, the voxel spacing was inherited directly from the image metadata (0.08 isometric) and a fixed bin width of 25 was used for the extraction process (the default setting used are listed in Supplementary Table 5: PyRadiomics settings).

### Blood biomarkers

The cell and plasma biomarker data set used as benchmarks in this study was from our previous work with all data and methods available in the publication^19^.

### Data analysis

Data wrangling, analysis and plotting was performed using R v4.1.2. Wrangling used the tidyverse v2.0.0 suite of packages^62^, and plots used the ggplot2 v3.5.1 suite of packages^63^. Zero variance, near zero variance (where the frequency of the most common value divided by the frequency of the second most common value exceeded 95, and the percentage of unique values was less than 10%) and highly correlating variables (set with a threshold of 0.9 Pearson’s coefficient) were used as feature filters using v1.0.10 of the recipes package from tidymodels^64^. Feature selection based on classification used the Boruta v8.0.0 package^20^ under default settings. Visualisation for parametric assumptions used the resid_panel function in the ggResidpanel v0.3.0 package^65^, and was performed after fitting the data to a linear model using the lm function in the stats v4.1.2 package^66^. Non parametric MANOVA was performed using PERMANOVA^22,23^ from the vegan v2.6-4 package. When doing multiple comparisons, a multiple comparison correction was applied using the false discovery rate (FDR) method^67^ implemented in the stats v4.1.2 package^66^. For pairwise performance metric analysis, Kruskal-Wallis^68^, Dunn’s^69^ and Wilcoxon^70^ methods were used and performed using the stats v4.1.2 package or v0.7.2 rstatix^71^ package and effect size using the v0.8.1 effsize^72^ package with p value FDR correction when doing multiple comparisons.

### Machine learning

Unsupervised machine learning used Uniform Manifold Approximation and Projection (UMAP) implemented using the umap v0.2.10.0^73^ package with default configuration parameters. Supervised machine learning was performed using a Random Forest learner^21^ from the ranger v0.16.0^74^ package implemented through the rand_forest function in tidymodels v1.2.0^28^. The total number of features considered at each split within a tree was set to three, each terminal node sample number was set to a minimum of five, and total number of trees in the forest was set to a thousand (remaining hyperparameters were set to the defaults, and are defined in Supplementary Table 6). Samples were balanced to a maximum of 1.2 to 1 (dominant class to subdominant class) ratio by sub-setting the dominant class samples randomly. Prediction performance was initially assessed on the training set (excluding four randomly selected samples from each class that were reserved for the final test set) using ten-fold cross-validation. In this procedure, one fold is held out as the test set while the remaining nine folds are combined to form the training set, and this process is repeated across all folds. To evaluate modelling variance, the entire cross-validation procedure was repeated three times. Accuracy, sensitivity, specificity, and Brier class were used as the performance metrics. The Kruskal Wallis and Dunn’s rank sum tests were used for difference assessments using the stats v4.1.2 package^66^ or v0.7.2 rstatix package^71^. In addition, a confusion matrix was made showing the percentage of truth for each class prediction (row-wise). To partition data into related clusters, PhenoGraph^26^ was used, which uses k-nearest neighbour graphs, Louvain community detection and density-based refinements to identify clusters, and was implemented with v0.99.1 of the RPhenograph^75^ package using the default settings. We initially evaluated the performance of Random Forest, XGBoost, radial SVM, and logistic regression (implemented using tidymodels v1.2.0^28^ and with hyperparameters listed in Supplementary Table 6) in classifying tumour types using radiomics data (Supplementary Fig. 7a). Statistical analyses (Supplementary Figs. 7b and 7c) revealed that logistic regression exhibited inferior performance, while the remaining learners showed no significant differences. Given its robust performance and widespread adoption, Random Forest was selected as the primary classifier for our analyses.

### Data imputation

Since samples from the cell, plasma and radiomic biomarker feature sets were not fully overlapping we imputed missing samples across the data sets so they could be combined to allow direct comparisons and to avoid sub-setting the data. Data was imputed using the Multivariate Imputation by Chained Equations (mice) v3.15.0 package^29^ where each imputation is predicted using a separate classification and regression tree (cart) which defines similar samples across the entire data structure for value imputation. Data was then restricted to 40 samples of each tumour class composed of all unimputed radiomic training samples (*n* = 35 for 4T1and *n* = 30 for CT26) and the remaining samples selected randomly from the partial imputed data across all the data sets (additional *n* = 5 for 4T1and *n* = 10 for CT26). To assess if sample numbers were adequate to reflect feature set potential predictive performance, learning curves were implemented^76^ (Supplementary Fig. 8). This analysis shows that model accuracy reaches the inflection point at ~ 20 samples across the three feature sets, beyond which the learning curve begins to plateau. This indicates that the current data sufficiently capture the underlying signal, and additional samples are expected to yield only marginal gains. Such saturation is consistent with theoretical and empirical observations of learning curves^76,77^, supporting the adequacy of the sample size for robust model development.

### Large language models

The large language models ChatGPT − 4o-mini (OpenAI, https://openai.com/chatgpt), and Claude v3.7 Sonnet (Anthropic, https://anthropic.com) were used to help with code writing and debugging, and for drafting the manuscript abstract.

## Supplementary Information

Below is the link to the electronic supplementary material.

Supplementary Material 1

Supplementary Material 2

Supplementary Material 3

Supplementary Material 4

## Data Availability

The initial (before filtering) radiomics features are available in Supplementary File (2) The raw sample data for the blood biomarkers are available in the original paper[19] and Supplementary File (1) The R code for the analysis is in Supplementary File (3) The tumour conformal contours and underlying DICOMS images are available in the Australian National University (ANU) DATA COMMONS repository at DOI: 10.25911/zkcm-ab43. Sphere contours and underlying DICOMS images are available in the Zenodo repository at DOI: 10.5281/zenodo.15070060.
