# Supplementary material for: Cancer classification with radiomics in controlled preclinical models
Source: Sci Rep. 2026 Jan 29;16:6647. doi: 10.1038/s41598-026-37757-8 (PMC12913988; doi:10.1038/s41598-026-37757-8)
Supplement: Supplementary file 4 — Supplementary Material 4 [file 41598_2026_37757_MOESM4_ESM.pdf]

Supplementary Information

Supplementary Table 1: Filtered radiomics feature characteristics

Feature	Shape	Intensity	Texture	Transformation
**original_gldm_DependenceVariance**	x	x	GLDM (Dependence Variance)	Original
**original_glszm_SmallAreaEmphasis**	x	x	GLSZM (Small Area Emphasis)	Original
**gradient_glcm_Correlation**	x	x	GLCM (Correlation)	Gradient
**gradient_glcm_MCC**	x	x	GLCM (MCC)	Gradient
**gradient_glcm_MaximumProbability**	x	x	GLCM (Max Probability)	Gradient
**gradient_gldm_SmallDependenceLowGrayLevelEmphasis**	x	x	GLDM (Small Dependence Low Gray Level Emphasis)	Gradient
**lbp-2D_glrlm_ShortRunLowGrayLevelEmphasis**	x	x	GLRLM (Short Run Low Gray Level Emphasis)	LBP
**logarithm_firstorder_Kurtosis**	x	First-order (Kurtosis)	x	Logarithm
**logarithm_glcm_ClusterProminence**	x	x	GLCM (Cluster Prominence)	Logarithm
**logarithm_gldm_LargeDependenceHighGrayLevelEmphasis**	x	x	GLDM (Large Dependence High Gray Level Emphasis)	Logarithm
**logarithm_gldm_SmallDependenceLowGrayLevelEmphasis**	x	x	GLDM (Small Dependence Low Gray Level Emphasis)	Logarithm
**logarithm_glszm_LargeAreaLowGrayLevelEmphasis**	x	x	GLSZM (Large Area Low Gray Level Emphasis)	Logarithm
**square_glcm_ClusterProminence**	x	x	GLCM (Cluster Prominence)	Square
**square_glszm_SizeZoneNonUniformityNormalized**	x	x	GLSZM (Size Zone Non-Uniformity Normalized)	Square
**squareroot_firstorder_Kurtosis**	x	First-order (Kurtosis)	x	Square Root
**wavelet-LHH_glcm_MCC**	x	x	GLCM (MCC)	Wavelet-LHH
**wavelet-HLL_glcm_ClusterShade**	x	x	GLCM (Cluster Shade)	Wavelet-HLL
**wavelet-LLL_glcm_MCC**	x	x	GLCM (MCC)	Wavelet-LLL

Acronyms used:

GLDM: Gray Level Dependence Matrix - Measures spatial relationships and dependencies in gray levels.

GLSZM: Gray Level Size Zone Matrix - Quantifies zones of similar gray levels in the ROI.

GLCM: Gray Level Co-occurrence Matrix - Captures texture by analyzing the spatial relationship of pixel pairs.

GLRLM: Gray Level Run Length Matrix - Measures the length of consecutive pixels with the same gray level.

LBP: Local Binary Pattern - Encodes texture patterns by comparing neighboring pixel intensities.

Wavelet: Multi-scale decomposition technique for capturing fine texture details.

LHH/HLL/HHL/LLL: Wavelet decompositions - Represent spatial frequency bands for texture analysis.

References:

1. Zwanenburg, A., Leger, S., Vallières, M., and Löck, S. (2016). Image biomarker standardisation initiative - feature definitions. In eprint arXiv:1612.07003 [cs.CV]

2. Aerts, H. J. W. L., et al. (2014). Decoding tumour phenotype by noninvasive imaging using a quantitative radiomics approach. Nature Communications, 5, 4006.

3. van Griethuysen, J. J. M., Fedorov, A., Parmar, C., Hosny, A., Aucoin, N., Narayan, V., Beets-Tan, R. G. H., Fillon-Robin, J. C., Pieper, S., Aerts, H. J. W. L. (2017). Computational Radiomics System to Decode the Radiographic Phenotype. Cancer Research, 77(21), e104–e107. `https://doi.org/10.1158/0008-5472.CAN-17-0339 <https://doi.org/10.1158/0008-5472.CAN-17-0339>`_

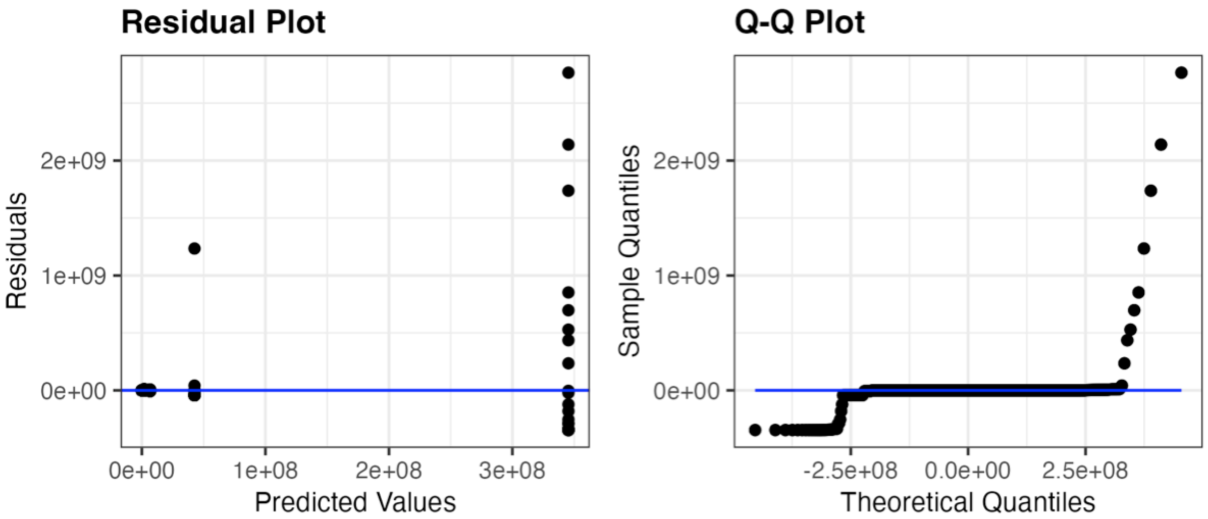

Supplementary Figure 1: Residual and Q-Q Plots modelled data
A linear model was created to estimate/predict how radiomic feature values depend on cancer class and radiomic feature interaction for statistical analysis. The difference between predicted values and the raw values (residuals) was plotted against predicted values in the Residual Plot (**first panel**) to see if the predicted values fit the raw data well. Higher predicted values had higher residuals suggesting non constant variance. The ordered residuals (sample quantiles) were plotted against the theoretical quantiles of a normally distributed population to form a Quantile- Quantile (Q-Q) plot (**second panel**) to assess distribution normality, which appeared absent.

Supplementary Table 2: Pairwise PERMANOVA with FDR p value (PrF) correction for multiple comparisons (Padj)

contrast	feature	Df	SumOfSqs	R2	F	PrF	Padj
4T1_vs_CT26	gradient_glcm_MCC	1	1.56e-01	0.316171180	32.8271538	0.001	0.00200000
4T1_vs_CT26	original_glszm_SmallAreaEmphasis	1	2.21e-02	0.258863510	24.7988184	0.001	0.00200000
4T1_vs_CT26	wavelet-HLL_glcm_ClusterShade	1	6.57e+03	0.235257791	21.8417435	0.001	0.00200000
4T1_vs_CT26	logarithm_gldm_LargeDependenceHighGrayLevelEmphasis	1	4.09e+14	0.213478122	19.2708520	0.001	0.00200000
4T1_vs_CT26	wavelet-LLL_glcm_MCC	1	1.22e-02	0.202661881	18.0462883	0.001	0.00200000
4T1_vs_CT26	gradient_glcm_Correlation	1	3.42e-01	0.188232667	16.4634850	0.001	0.00200000
4T1_vs_CT26	square_glcm_ClusterProminence	1	9.62e+05	0.181514667	15.7455984	0.001	0.00200000
4T1_vs_CT26	square_glszm_SizeZoneNonUniformityNormalized	1	2.04e-03	0.139391764	11.4997915	0.001	0.00200000
4T1_vs_CT26	logarithm_glszm_LargeAreaLowGrayLevelEmphasis	1	7.14e+06	0.112257760	8.9781702	0.001	0.00200000
4T1_vs_CT26	wavelet-LHH_glcm_MCC	1	1.56e-03	0.103778678	8.2215029	0.003	0.00540000
4T1_vs_CT26	gradient_glcm_MaximumProbability	1	1.49e-07	0.089780212	7.0031383	0.014	0.01938462
4T1_vs_CT26	logarithm_firstorder_Kurtosis	1	1.08e+07	0.079670076	6.1462474	0.011	0.01800000
4T1_vs_CT26	logarithm_glcm_ClusterProminence	1	1.66e+18	0.071345516	5.4547000	0.014	0.01938462
4T1_vs_CT26	logarithm_gldm_SmallDependenceLowGrayLevelEmphasis	1	1.93e-08	0.064113402	4.8638923	0.019	0.02442857
4T1_vs_CT26	squareroot_firstorder_Kurtosis	1	5.82e+03	0.062581028	4.7398795	0.036	0.04320000
4T1_vs_CT26	gradient_gldm_SmallDependenceLowGrayLevelEmphasis	1	1.20e-07	0.035730865	2.6308956	0.112	0.12600000
4T1_vs_CT26	original_gldm_DependenceVariance	1	1.07e-01	0.016730859	1.2081036	0.287	0.30388235
4T1_vs_CT26	lbp-2D_glrlm_ShortRunLowGrayLevelEmphasis	1	3.32e-05	0.009386197	0.6727344	0.468	0.46800000

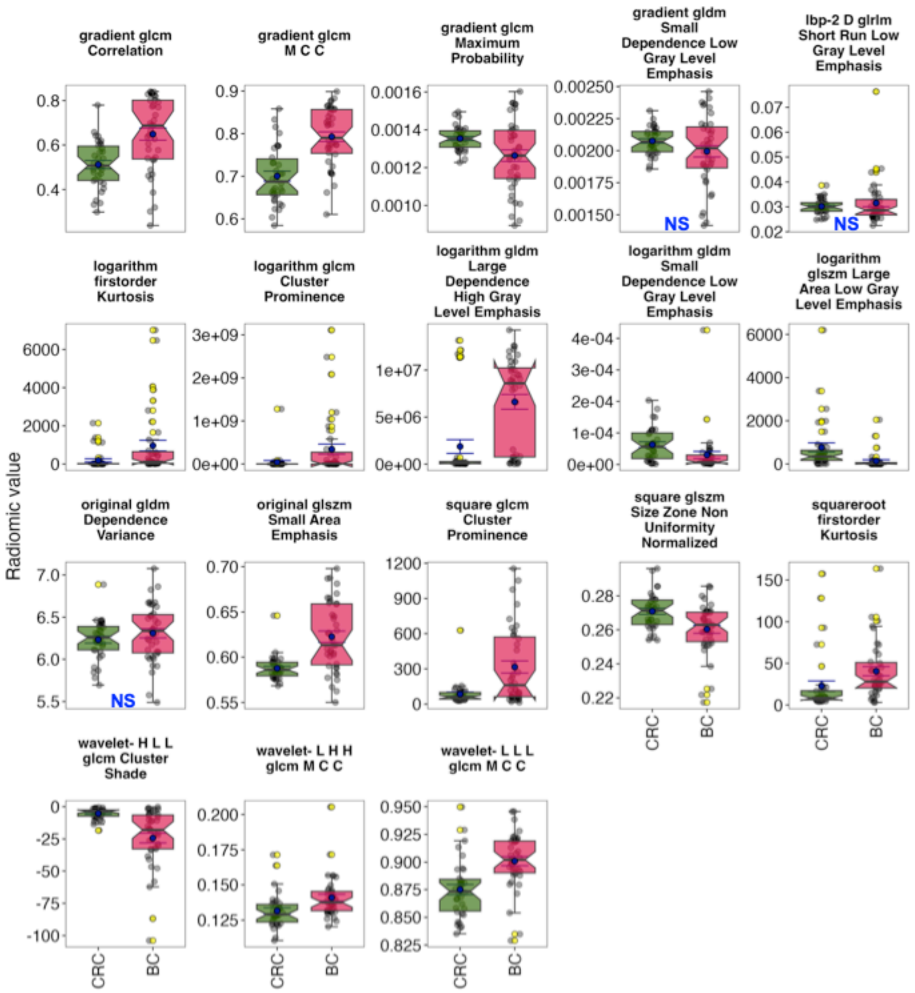

Supplementary Figure 2: Comparison of selected radiomic features derived from CRC and BC images

The eighteen filtered radiomic features derived from CT26 CRC and 4T1 BC tumour micro-CT images (as described in Figures 1 and 2) had their values plotted for each tumour type. Plots are boxplots with notches representing 95% confidence intervals, blue points showing means, error bars (inner whiskers) being standard error of means, yellow points as outliers, and grey points individual sample values (CT26 samples = 34 and 4T1samples =39). Features that were not significantly different based on the analysis described in Supplementary Table 2 are noted as **NS.**

Supplementary Table 3: Blood cell, plasma protein and radiomic biomarkers used for machine learning

Number	Blood cell biomarkers	Plasma biomarkers	Radiomic biomarkers	Radiomic truncated names
1	B cells	CXCL1 (1)	original_gldm_DependenceVariance	ori_gld_Depe
2	CD4 T cells	TGF-b1	original_glszm_SmallAreaEmphasis	ori_gls_Smal
3	Tregs	IL-18	gradient_glcm_Correlation	gra_glc_Corr
4	CD8 T cells	IL-23	gradient_glcm_MCC	gra_glc_MCC
5	CD8 T cells Ly6C+	CCL22 (1)	gradient_glcm_MaximumProbability	gra_glc_Maxi
6	NK cells	IL-10	gradient_gldm_SmallDependenceLowGrayLevelEmphasis	gra_gld_Smal
7	NK cells Ly6C+	IL-12p70	lbp-2D_glrlm_ShortRunLowGrayLevelEmphasis	lbp_2D_glr_Shor
8	Eosinophils	IL-6	logarithm_firstorder_Kurtosis	log_fir_Kurt
9	Macrophage	TNF-a	logarithm_glcm_ClusterProminence	log_glc_Clus
10	DC	G-CSF	logarithm_gldm_LargeDependenceHighGrayLevelEmphasis	log_gld_Larg
11	DC PD-L1+	CCL17 (1)	logarithm_gldm_SmallDependenceLowGrayLevelEmphasis	log_gld_Smal
12	CD11b+ Ly6C_G-	IL-12p40	logarithm_glszm_LargeAreaLowGrayLevelEmphasis	log_gls_Larg
13	CD11b+ Ly6C_G- PD-L1+	IL-1b	square_glcm_ClusterProminence	squ_glc_Clus
14	CD11b+ Ly6Cint Ly6G-	CCL5	square_glszm_SizeZoneNonUniformityNormalized	squ_gls_Size
15	Monocytes	CCL20	squareroot_firstorder_Kurtosis	squ_fir_Kurt
16	Neutrophils	CCL11	wavelet-LHH_glcm_MCC	wav_LHH_glc_MCC
17		CCL17 (2)	wavelet-HLL_glcm_ClusterShade	wav_HLL_glc_Clus
18		CXCL1 (2)	wavelet-LLL_glcm_MCC	wav_LLL_glc_MCC
19		CCL2		
20		CCL4		
21		CXCL13		
22		CXCL5		
23		CCL22 (2)		

(1) and (2) are molecules detected with different antibody clones

Blood cell and plasma protein data has been normalised to controls as fold changes as described in the original paper below

Simon Davis, D. A. et al. Machine learning predicts cancer subtypes and progression from blood immune signatures. PLoS One 17, e0264631 (2022)

Supplementary Table 4: Sample numbers used in the training data sets

Dataset	Imputation_Status	Majority_Class	Minority_Class	Original_Majority_Count	Original_Minority_Count	Original_Ratio	New_Majority_Count	New_Minority_Count	New_Ratio
Cells	Unimputed	4T1	CT26	152	92	1.652174	111	92	1.206522
Cells	Imputed	4T1	CT26	169	97	1.742268	117	97	1.206186
Plasma	Unimputed	4T1	CT26	151	92	1.641304	111	92	1.206522
Plasma	Imputed	4T1	CT26	169	97	1.742268	117	97	1.206186
Radiomics	Unimputed	4T1	CT26	35	30	1.166667	35	30	1.166667
Radiomics	Imputed	4T1	CT26	169	97	1.742268	117	97	1.206186
Radiomics sphere		4T1	CT26	42	30	1.400000	36	30	1.200000
Radiomics non tumour sphere		4T1	CT26	35	20	1.750000	24	20	1.200000
Radiomics MONAI		4T1	CT26	38	30	1.266667	36	30	1.200000

Ratios are majority cancer sample number to minority cancer sample number

Sphere and MONAI contour are unimputed

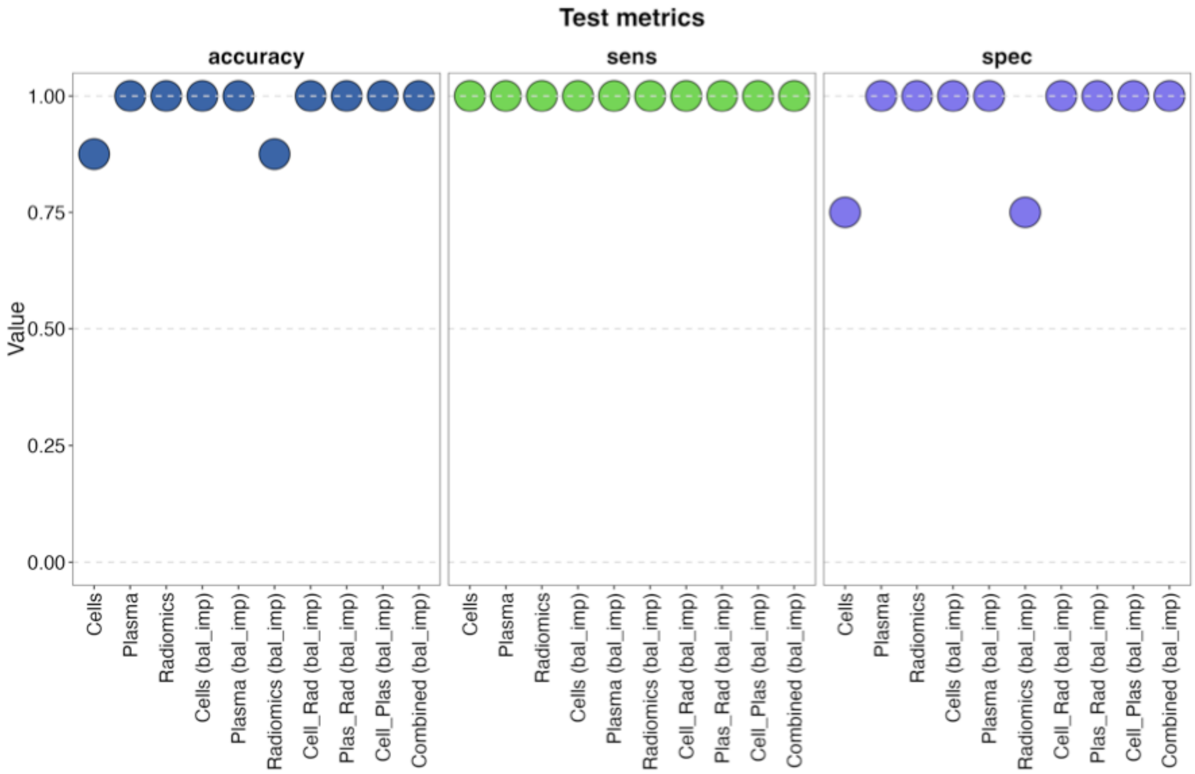

Supplementary Figure 3: Prediction of cancer class using cell, plasma and radiomic feature on a single common test data set

Random Forest models were trained on training data (generated as described in Figure 5) from blood cell, plasma protein and radiomics features. Models were then used to predict cancer class from a test set of the same four samples from each class. The accuracy, sensitivity and specificity were graphed as points.

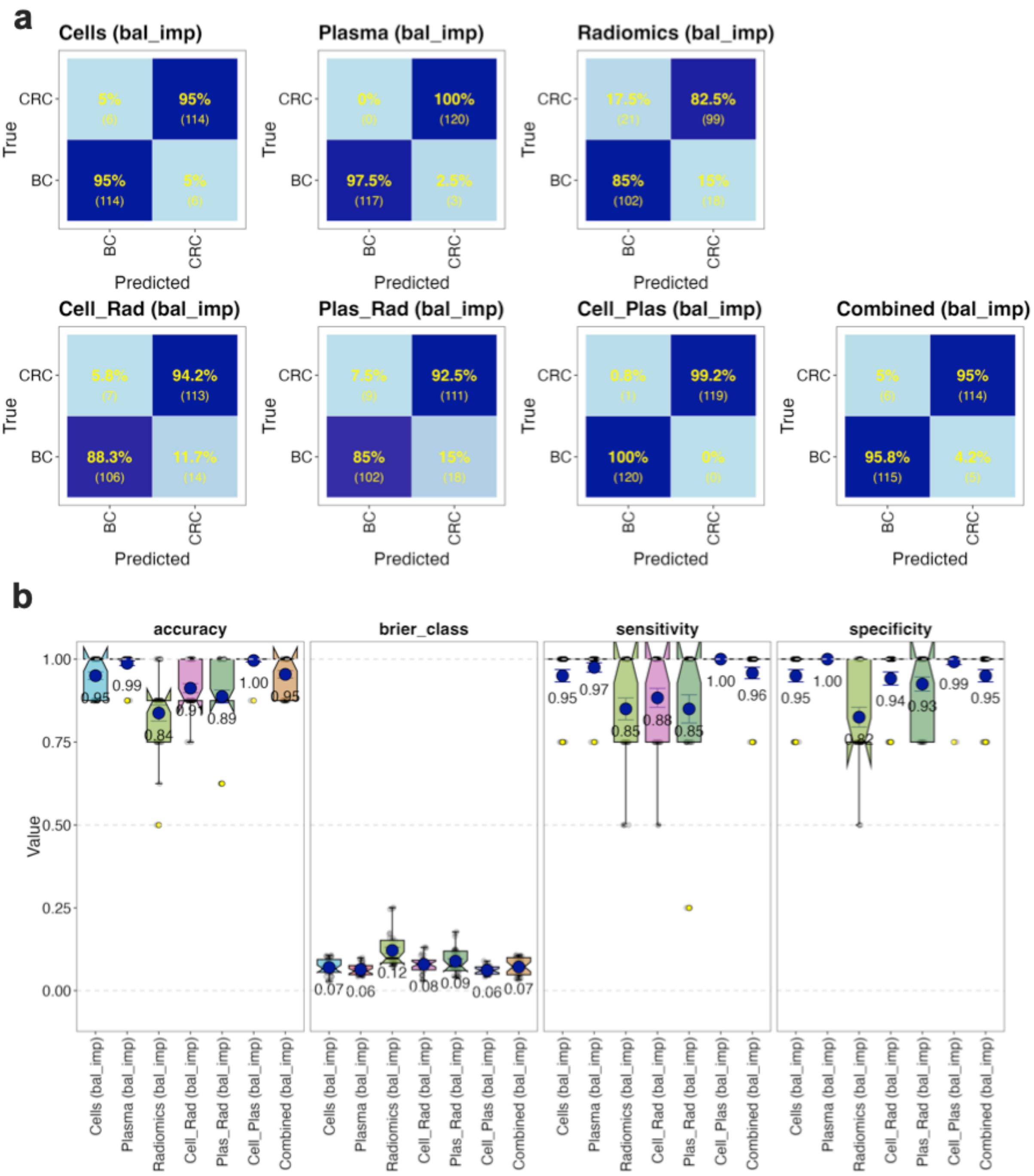
a
Cells (bal_imp)
Plasma (bal_imp)
Radiomics (bal_imp)
Cell_Rad (bal_imp)
Plas_Rad (bal_imp)
Cell_Plas (bal_imp)
Combined (bal_imp)
True
Predicted
CRC
BC
b
accuracy
brier_class
sensitivity
specificity
Value

c

metric	Test	Comparison	Effect Size	P-value	Adjusted P-value	Significance
accuracy	Kruskal-Wallis	All	χ^2 = 69.451	0.000	N/A	***
	Wilcoxon	Cells (bal_imp) vs Plasma (bal_imp)	Cliff's Δ = -0.300	0.008	0.014	*
	Wilcoxon	Cells (bal_imp) vs Radiomics (bal_imp)	Cliff's Δ = 0.533	< 0.001	< 0.001	***
	Wilcoxon	Cells (bal_imp) vs Cell_Rad (bal_imp)	Cliff's Δ = 0.260	0.050	0.062	ns
	Wilcoxon	Cells (bal_imp) vs Plas_Rad (bal_imp)	Cliff's Δ = 0.340	0.011	0.018	*
	Wilcoxon	Cells (bal_imp) vs Cell_Plas (bal_imp)	Cliff's Δ = -0.367	< 0.001	0.002	**
	Wilcoxon	Cells (bal_imp) vs Combined (bal_imp)	Cliff's Δ = -0.033	0.799	0.799	ns
	Wilcoxon	Plasma (bal_imp) vs Radiomics (bal_imp)	Cliff's Δ = 0.733	< 0.001	< 0.001	***
	Wilcoxon	Plasma (bal_imp) vs Cell_Rad (bal_imp)	Cliff's Δ = 0.540	< 0.001	< 0.001	***
	Wilcoxon	Plasma (bal_imp) vs Plas_Rad (bal_imp)	Cliff's Δ = 0.610	< 0.001	< 0.001	***
	Wilcoxon	Plasma (bal_imp) vs Cell_Plas (bal_imp)	Cliff's Δ = -0.067	0.313	0.346	ns
	Wilcoxon	Plasma (bal_imp) vs Combined (bal_imp)	Cliff's Δ = 0.267	0.016	0.023	*
	Wilcoxon	Radiomics (bal_imp) vs Cell_Rad (bal_imp)	Cliff's Δ = -0.331	0.016	0.023	*
	Wilcoxon	Radiomics (bal_imp) vs Plas_Rad (bal_imp)	Cliff's Δ = -0.237	0.086	0.100	ns
	Wilcoxon	Radiomics (bal_imp) vs Cell_Plas (bal_imp)	Cliff's Δ = -0.778	< 0.001	< 0.001	***
	Wilcoxon	Radiomics (bal_imp) vs Combined (bal_imp)	Cliff's Δ = -0.556	< 0.001	< 0.001	***
	Wilcoxon	Cell_Rad (bal_imp) vs Plas_Rad (bal_imp)	Cliff's Δ = 0.090	0.499	0.524	ns
	Wilcoxon	Cell_Rad (bal_imp) vs Cell_Plas (bal_imp)	Cliff's Δ = -0.602	< 0.001	< 0.001	***
	Wilcoxon	Cell_Rad (bal_imp) vs Combined (bal_imp)	Cliff's Δ = -0.291	0.028	0.037	*
	Wilcoxon	Plas_Rad (bal_imp) vs Cell_Plas (bal_imp)	Cliff's Δ = -0.670	< 0.001	< 0.001	***
	Wilcoxon	Plas_Rad (bal_imp) vs Combined (bal_imp)	Cliff's Δ = -0.370	0.006	0.011	*
	Wilcoxon	Cell_Plas (bal_imp) vs Combined (bal_imp)	Cliff's Δ = 0.333	0.001	0.003	**
brier_class	Kruskal-Wallis	All	χ^2 = 46.872	0.000	N/A	***
	Wilcoxon	Cells (bal_imp) vs Plasma (bal_imp)	Cliff's Δ = 0.182	0.228	0.320	ns
	Wilcoxon	Cells (bal_imp) vs Radiomics (bal_imp)	Cliff's Δ = -0.664	< 0.001	< 0.001	***
	Wilcoxon	Cells (bal_imp) vs Cell_Rad (bal_imp)	Cliff's Δ = -0.196	0.196	0.294	ns
	Wilcoxon	Cells (bal_imp) vs Plas_Rad (bal_imp)	Cliff's Δ = -0.267	0.077	0.144	ns
	Wilcoxon	Cells (bal_imp) vs Cell_Plas (bal_imp)	Cliff's Δ = 0.209	0.167	0.270	ns
	Wilcoxon	Cells (bal_imp) vs Combined (bal_imp)	Cliff's Δ = -0.040	0.796	0.880	ns
	Wilcoxon	Plasma (bal_imp) vs Radiomics (bal_imp)	Cliff's Δ = -0.836	< 0.001	< 0.001	***
	Wilcoxon	Plasma (bal_imp) vs Cell_Rad (bal_imp)	Cliff's Δ = -0.356	0.018	0.043	*
	Wilcoxon	Plasma (bal_imp) vs Plas_Rad (bal_imp)	Cliff's Δ = -0.327	0.030	0.064	ns
	Wilcoxon	Plasma (bal_imp) vs Cell_Plas (bal_imp)	Cliff's Δ = 0.024	0.877	0.920	ns
	Wilcoxon	Plasma (bal_imp) vs Combined (bal_imp)	Cliff's Δ = -0.151	0.318	0.393	ns
	Wilcoxon	Radiomics (bal_imp) vs Cell_Rad (bal_imp)	Cliff's Δ = 0.531	< 0.001	0.002	**
	Wilcoxon	Radiomics (bal_imp) vs Plas_Rad (bal_imp)	Cliff's Δ = 0.409	0.007	0.020	*
	Wilcoxon	Radiomics (bal_imp) vs Cell_Plas (bal_imp)	Cliff's Δ = 0.940	< 0.001	< 0.001	***
	Wilcoxon	Radiomics (bal_imp) vs Combined (bal_imp)	Cliff's Δ = 0.576	< 0.001	< 0.001	***
	Wilcoxon	Cell_Rad (bal_imp) vs Plas_Rad (bal_imp)	Cliff's Δ = -0.016	0.923	0.923	ns
	Wilcoxon	Cell_Rad (bal_imp) vs Cell_Plas (bal_imp)	Cliff's Δ = 0.447	0.003	0.011	*
	Wilcoxon	Cell_Rad (bal_imp) vs Combined (bal_imp)	Cliff's Δ = 0.133	0.379	0.442	ns
	Wilcoxon	Plas_Rad (bal_imp) vs Cell_Plas (bal_imp)	Cliff's Δ = 0.364	0.016	0.041	*
	Wilcoxon	Plas_Rad (bal_imp) vs Combined (bal_imp)	Cliff's Δ = 0.262	0.082	0.144	ns
	Wilcoxon	Cell_Plas (bal_imp) vs Combined (bal_imp)	Cliff's Δ = -0.176	0.246	0.323	ns
sensitivity	Kruskal-Wallis	All	χ^2 = 31.290	0.000	N/A	***
	Wilcoxon	Cells (bal_imp) vs Plasma (bal_imp)	Cliff's Δ = -0.100	0.287	0.377	ns
	Wilcoxon	Cells (bal_imp) vs Radiomics (bal_imp)	Cliff's Δ = 0.293	0.019	0.044	*
	Wilcoxon	Cells (bal_imp) vs Cell_Rad (bal_imp)	Cliff's Δ = 0.213	0.077	0.114	ns
	Wilcoxon	Cells (bal_imp) vs Plas_Rad (bal_imp)	Cliff's Δ = 0.220	0.069	0.111	ns
	Wilcoxon	Cells (bal_imp) vs Cell_Plas (bal_imp)	Cliff's Δ = -0.200	0.011	0.029	*
	Wilcoxon	Cells (bal_imp) vs Combined (bal_imp)	Cliff's Δ = -0.033	0.749	0.787	ns
	Wilcoxon	Plasma (bal_imp) vs Radiomics (bal_imp)	Cliff's Δ = 0.380	0.001	0.007	**
	Wilcoxon	Plasma (bal_imp) vs Cell_Rad (bal_imp)	Cliff's Δ = 0.307	0.007	0.024	*
	Wilcoxon	Plasma (bal_imp) vs Plas_Rad (bal_imp)	Cliff's Δ = 0.310	0.006	0.024	*
	Wilcoxon	Plasma (bal_imp) vs Cell_Plas (bal_imp)	Cliff's Δ = -0.100	0.081	0.114	ns
	Wilcoxon	Plasma (bal_imp) vs Combined (bal_imp)	Cliff's Δ = 0.067	0.459	0.567	ns
	Wilcoxon	Radiomics (bal_imp) vs Cell_Rad (bal_imp)	Cliff's Δ = -0.089	0.509	0.593	ns
	Wilcoxon	Radiomics (bal_imp) vs Plas_Rad (bal_imp)	Cliff's Δ = -0.060	0.659	0.728	ns
	Wilcoxon	Radiomics (bal_imp) vs Cell_Plas (bal_imp)	Cliff's Δ = -0.467	< 0.001	< 0.001	***
	Wilcoxon	Radiomics (bal_imp) vs Combined (bal_imp)	Cliff's Δ = -0.322	0.009	0.026	*
	Wilcoxon	Cell_Rad (bal_imp) vs Plas_Rad (bal_imp)	Cliff's Δ = 0.020	0.885	0.885	ns
	Wilcoxon	Cell_Rad (bal_imp) vs Cell_Plas (bal_imp)	Cliff's Δ = -0.400	< 0.001	< 0.001	***
	Wilcoxon	Cell_Rad (bal_imp) vs Combined (bal_imp)	Cliff's Δ = -0.244	0.039	0.069	ns
	Wilcoxon	Plas_Rad (bal_imp) vs Cell_Plas (bal_imp)	Cliff's Δ = -0.400	< 0.001	< 0.001	***
	Wilcoxon	Plas_Rad (bal_imp) vs Combined (bal_imp)	Cliff's Δ = -0.250	0.035	0.068	ns
	Wilcoxon	Cell_Plas (bal_imp) vs Combined (bal_imp)	Cliff's Δ = 0.167	0.021	0.045	*
specificity	Kruskal-Wallis	All	χ^2 = 41.995	0.000	N/A	***
	Wilcoxon	Cells (bal_imp) vs Plasma (bal_imp)	Cliff's Δ = -0.200	0.011	0.023	*
	Wilcoxon	Cells (bal_imp) vs Radiomics (bal_imp)	Cliff's Δ = 0.420	0.001	0.005	**
	Wilcoxon	Cells (bal_imp) vs Cell_Rad (bal_imp)	Cliff's Δ = 0.033	0.764	0.802	ns
	Wilcoxon	Cells (bal_imp) vs Plas_Rad (bal_imp)	Cliff's Δ = 0.100	0.380	0.470	ns
	Wilcoxon	Cells (bal_imp) vs Cell_Plas (bal_imp)	Cliff's Δ = -0.167	0.048	0.071	ns
	Wilcoxon	Cells (bal_imp) vs Combined (bal_imp)	Cliff's Δ = 0.000	1.000	1.000	ns
	Wilcoxon	Plasma (bal_imp) vs Radiomics (bal_imp)	Cliff's Δ = 0.600	< 0.001	< 0.001	***
	Wilcoxon	Plasma (bal_imp) vs Cell_Rad (bal_imp)	Cliff's Δ = 0.233	0.005	0.016	*
	Wilcoxon	Plasma (bal_imp) vs Plas_Rad (bal_imp)	Cliff's Δ = 0.300	0.001	0.005	**
	Wilcoxon	Plasma (bal_imp) vs Cell_Plas (bal_imp)	Cliff's Δ = 0.033	0.334	0.467	ns
	Wilcoxon	Plasma (bal_imp) vs Combined (bal_imp)	Cliff's Δ = 0.200	0.011	0.023	*
	Wilcoxon	Radiomics (bal_imp) vs Cell_Rad (bal_imp)	Cliff's Δ = -0.390	0.003	0.010	**
	Wilcoxon	Radiomics (bal_imp) vs Plas_Rad (bal_imp)	Cliff's Δ = -0.330	0.013	0.024	*
	Wilcoxon	Radiomics (bal_imp) vs Cell_Plas (bal_imp)	Cliff's Δ = -0.570	< 0.001	< 0.001	***
	Wilcoxon	Radiomics (bal_imp) vs Combined (bal_imp)	Cliff's Δ = -0.420	0.001	0.005	**
	Wilcoxon	Cell_Rad (bal_imp) vs Plas_Rad (bal_imp)	Cliff's Δ = 0.067	0.569	0.664	ns
	Wilcoxon	Cell_Rad (bal_imp) vs Cell_Plas (bal_imp)	Cliff's Δ = -0.200	0.025	0.043	*
	Wilcoxon	Cell_Rad (bal_imp) vs Combined (bal_imp)	Cliff's Δ = -0.033	0.764	0.802	ns
	Wilcoxon	Plas_Rad (bal_imp) vs Cell_Plas (bal_imp)	Cliff's Δ = -0.267	0.006	0.016	*
	Wilcoxon	Plas_Rad (bal_imp) vs Combined (bal_imp)	Cliff's Δ = -0.100	0.380	0.470	ns
	Wilcoxon	Cell_Plas (bal_imp) vs Combined (bal_imp)	Cliff's Δ = 0.167	0.048	0.071	ns

Supplementary Figure 4: Cancer type predictions using combinations of feature sets
Cell, plasma and radiomic data sets were combined and missing data imputed using classification and regression trees in Multivariate Imputation by Chained Equations[29]. Data was then balanced to 40 samples of each tumour class composed of all unimputed radiomic training samples (n = 35 for 4T1and n = 30 for CT26) and the remaining samples randomly selected from the partial imputed data across all the data sets (n = 5 for 4T1and n = 10 for CT26, see Supplementary Table 4, imputed data, for number details). Random Forest was then used to assess if BC and CRC classification improved when combining features derived from blood cells, plasma proteins and/or radiomics using repeated CV as described in Figure 5. CV predictions of BC and CRC were depicted as confusion matrices (**a**) and performance metrics (**b**) as described in Figure 5. Statistical analysis of overall differences (by Kruskal-Wallis test) and pairwise comparisons (by Wilcoxon test) for each feature type for each metric is also displayed (**c**). Adjusted P-values use FDR correction. Red arrows highlight the impact of radiomic features in some key comparisons. Adjusted P-values use FDR correction. The significance column is based on adjusted P-value (p.adj) thresholds: ns = not significant ($p.adj \geq 0.05$), * = $p.adj < 0.05$, ** = $p.adj < 0.01$, and *** = $p.adj < 0.001$.

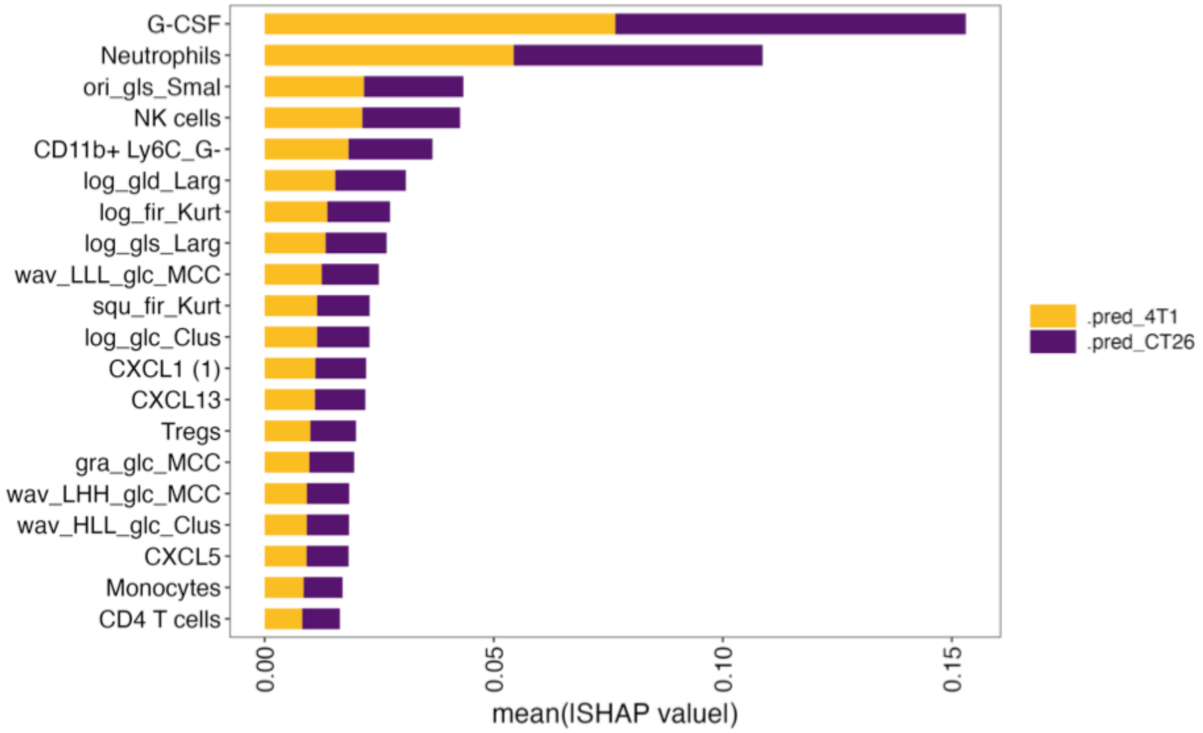

Supplementary Figure 5: Importance of different feature types in cancer classification
Cell, plasma and radiomic data sets were combined, missing data imputed and balanced (n = 40 for 4T1and n = 40 for CT26) as described in Supplementary Figure 3. Random Forest was then used to model BC and CRC classification. Shapley Additive exPlanations (SHAP)[49] was then used to assess the contribution of each feature to the predictions made by the Random Forest classifier. Plotted are the mean absolute SHAP values for the top twenty ranked features for predicting both 4T1 and CT26 as composition bar plots. The radiomic features are listed as truncated feature names described in Supplementary Table 3.

a

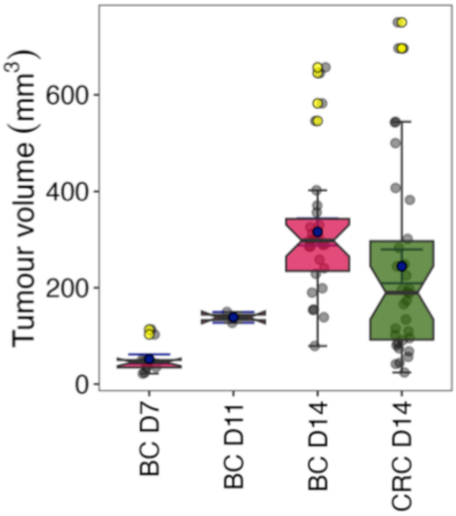

b

Tumour_Model	n_replicates	Ages	n_samples	Total
4T1	1	D7	5	39
	1	D11	2	
	1	D14	22	
	2	D14, D7	5	
CT26	1	D14	34	34

n_replicates = times an animal was sampled in an experiment

Ages = tumour growth days at scan

n_samples = number of animals with that replicate pattern

Total = total sample instances per tumour model (includes animals scanned multiple times; see n_replicates and Ages i.e. five 4T1 animals were scanned twice)

Supplementary Figure 6: CRC and BC tumour sizes, and sample number breakdown CT26 CRC and 4T1 BC tumours were established subcutaneously in the right hind flank of BALB/c female mice and left to grow for 7 – 14 days. Tumour contour volumes from radiomic features of the two classes across the imaging time points are shown in **a**. Plots in **a** are boxplots with notches representing 95% confidence intervals, blue points showing means, error bars (inner whiskers) being standard error of means, yellow points as outliers, and grey points individual sample values (CT26 samples = 34 and 4T1 samples = 39). Numbers of animals scanned at the time points for analysis show precise times animal were used within experiments (n_replicates), times they were scanned post tumour establishment (Age), numbers of animals with duplicate or single scans (n_sample), and total sample instances per tumour model (Total) b.

Supplementary Table 5: PyRadiomics settings

Setting (PyRadiomics default)	Default value	Description
normalize	False	Whether to normalize before resampling
normalizeScale	1	Scale after normalization
removeOutliers	None	Outlier removal threshold (σ units)
resampledPixelSpacing	None	Voxel spacing for resampling (None = no resampling)
interpolator	sitkBSpline	Interpolator for resampling images
padDistance	5	Padding voxels around ROI before resampling
distances	[1]	Texture neighborhood distance(s)
force2D	False	Force 2D texture calc
force2Ddimension	0	Dimension defining 2D slices (if force2D=TRUE)
resegmentRange	None	Gray-value resegmentation range
label	1	Mask label value for ROI
additionalInfo	True	Include provenance / metadata in output
binWidth	25	Bin width for gray-level discretization
enableAllImageTypes()	Method call	Enables and applies all accepted filters in PyRadiomics
enableAllFeatures()	Method call	Enables and applies all accepted feature calculations in PyRadiomics
voxelBased	False	Performs segmentation-based extraction
minimunROISize	None	Minimum size limit for the ROI label (None = no limit)
minimumROIDimensions	2	ROI must be at least 2D

Supplementary Table 6: Machine learning learner hyperparameters

Model†	Hyperparameter	Value	Default
Random Forest	mtry	3	sqrt(predictors) if classification
Random Forest	trees	1000	500
Random Forest	min_n	5	1
Random Forest	splitrule	gini	gini (classification), variance (regression)
XGBoost	trees	1000	100
XGBoost	tree_depth	6	6
XGBoost	learn_rate	0.1	0.3
XGBoost	loss_reduction (gamma)	0	0
XGBoost	min_child_weight	Default	1
XGBoost	subsample	Default	1
XGBoost	colsample_bytree	Default	1
Logistic Regression	penalty	Not set	0 (no regularisation)
Logistic Regression	mixture	Not set	N/A (glm has no mixture parameter)
Logistic Regression	link	logit	logit
SVM (Radial)	cost	1	1
SVM (Radial)	rbf_sigma	0.1	1 / n_features
SVM (Radial)	kernel	radial basis	radial basis
SVM (Radial)	prob.model	TRUE (via parsnip)	FALSE (kernlab default)

†Defaults are based on tidymodels v1.2.0 (parsnip) with ranger v0.16.0, xgboost v1.7+, kernlab v0.9-32, and glm from the base R stats package.

a

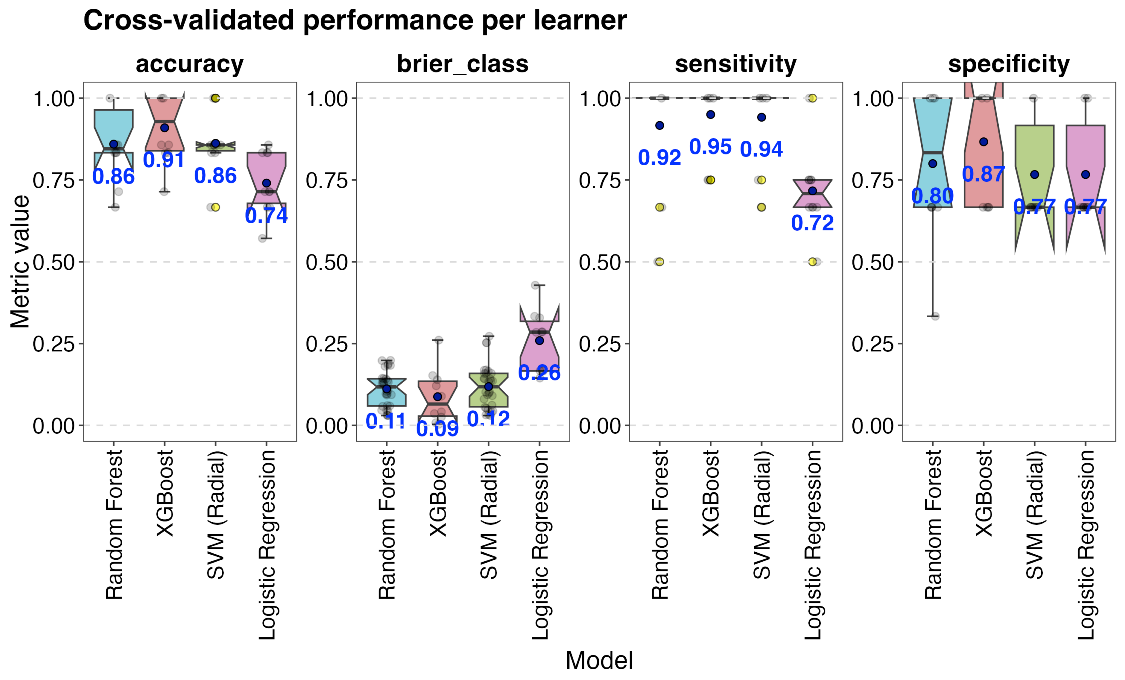

b

.metric	n	statistic	df	p	method
accuracy	40	11.545308	3	0.009110	Kruskal-Wallis
brier_class	80	20.763453	3	0.000118	Kruskal-Wallis
sensitivity	40	14.671498	3	0.002120	Kruskal-Wallis
specificity	40	2.250725	3	0.522000	Kruskal-Wallis

Wilcoxon rank-sum test, two-sided

c

.metric	group1	group2	n1	n2	statistic	p	p.adj	p.adj.signif
accuracy	Logistic Regression	SVM (Radial)	10	10	2.530522	0.01138929045	0.0341678713	*
accuracy	Logistic Regression	XGBoost	10	10	3.207290	0.00133992007	0.0080395204	**
brier_class	Logistic Regression	Random Forest	10	30	-3.877325	0.00010561128	0.0003168338	***
brier_class	Logistic Regression	SVM (Radial)	10	30	-3.739831	0.00018414394	0.0003682879	***
brier_class	Logistic Regression	XGBoost	10	10	-4.205059	0.00002610139	0.0001566084	***
sensitivity	Logistic Regression	Random Forest	10	10	2.909266	0.00362278769	0.0072455754	**
sensitivity	Logistic Regression	SVM (Radial)	10	10	3.141565	0.00168047771	0.0050414331	**
sensitivity	Logistic Regression	XGBoost	10	10	3.285369	0.00101849056	0.0050414331	**

Dunn's posthoc test with Benjamini–Hochberg adjustment, alpha = 0.05. Non-significant results (ns) excluded.

Supplementary Figure 7: Different learner performance using radiomics in tumour classification

Cell, plasma and radiomic (using the filtered 18 radiomics features) data sets were combined and missing data imputed using classification and regression trees in Multivariate Imputation by Chained Equations[29]. Data was then balanced to 40 samples of each tumour class composed of all unimputed radiomic training samples (n = 35 for 4T1 and n = 30 for CT26) and the remaining samples randomly selected from the partial imputed data across all the data sets (n = 5 for 4T1 and n = 10 for CT26, see Supplementary Table 4, imputed data, for number details). Random Forest was then used to assess if BC and CRC classification improved when using the radiomics data when using other learners, namely, XGBoost, SVM (Radial) and Logistic Regression using repeated CV as described in Figure 5. CV predictions of BC and CRC were depicted as performance metrics (**a**) as described in Figure 5.

Statistical analysis of overall differences (by Kruskal-Wallis test **b**) and pairwise comparisons (by Dunn's test **c**) for each learner for each metric is also displayed (**c**). Adjusted P-values use FDR (Benjamini–Hochberg) correction. The significance column is based on adjusted P-value (p.adj) thresholds: ns = not significant (p.adj ≥ 0.05; not shown), * = p.adj < 0.05, ** = p.adj < 0.01, and *** = p.adj < 0.001.

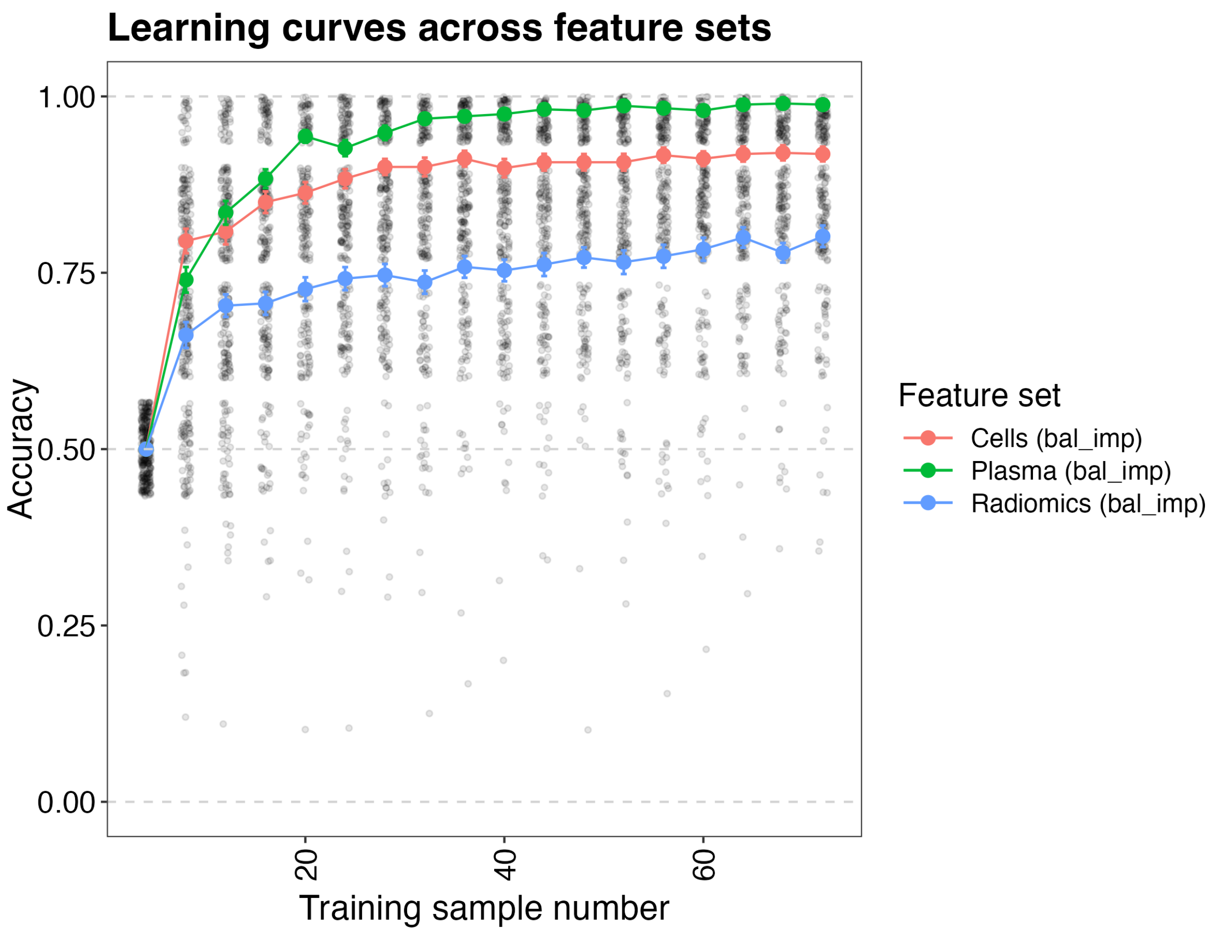

Supplementary Figure 8: Learning curve performance across the data sets
Cell, plasma and radiomic (using the filtered 18 radiomics features) data sets were combined and missing data imputed using classification and regression trees in Multivariate Imputation by Chained Equations[29]. Data was then balanced to 65 samples of each tumour class composed of all unimputed radiomic training samples (n = 35 for 4T1 and n = 30 for CT26) and the remaining samples randomly selected from the partial imputed data across all the data sets (n = 5 for 4T1 and n = 10 for CT26, see Supplementary Table 4, imputed data, for number details). Random Forest models were used to assess BC and CRC classification accuracy as the training sample size increased on a fixed test set. The test set comprised three samples per class randomly selected from the data. From the remaining data the training set was incrementally increased in even numbers, balanced by class. The procedure was repeated 100 times to cover the dataset. All replicate accuracy measurements are shown (light grey points), with mean accuracies (large points) and standard errors of the mean (error bars) indicated for each training sample size.
